# Protein shape sampled by ion mobility mass spectrometry consistently improves protein structure prediction

**DOI:** 10.1038/s41467-022-32075-9

**Published:** 2022-07-28

**Authors:** SM Bargeen Alam Turzo, Justin T. Seffernick, Amber D. Rolland, Micah T. Donor, Sten Heinze, James S. Prell, Vicki H. Wysocki, Steffen Lindert

**Affiliations:** 1 Department of Chemistry and Biochemistry and Resource for Native Mass Spectrometry Guided Structural Biology, Ohio State University, Columbus, OH 43210 USA; 2 Department of Chemistry and Biochemistry and Materials Science Institute, University of Oregon, Eugene, OR 97403 USA

**Keywords:** Molecular modelling, Computational biophysics, Protein structure predictions, Mass spectrometry

## Abstract

Ion mobility (IM) mass spectrometry provides structural information about protein shape and size in the form of an orientationally-averaged collision cross-section (CCS_IM_). While IM data have been used with various computational methods, they have not yet been utilized to predict monomeric protein structure from sequence. Here, we show that IM data can significantly improve protein structure determination using the modelling suite Rosetta. We develop the Rosetta Projection Approximation using Rough Circular Shapes (PARCS) algorithm that allows for fast and accurate prediction of CCS_IM_ from structure. Following successful testing of the PARCS algorithm, we use an integrative modelling approach to utilize IM data for protein structure prediction. Additionally, we propose a confidence metric that identifies near native models in the absence of a known structure. The results of this study demonstrate the ability of IM data to consistently improve protein structure prediction.

## Abstract (web summary)

Collision cross sections (CCS) from ion mobility mass spectrometry provide information about protein shape and size. Here, the authors develop an algorithm to predict CCS and integrate experimental ion mobility data into Rosetta-based molecular modelling to predict protein structures from sequence.

## Introduction

Proteins are at the core of virtually all cellular processes. Therefore, comprehensive knowledge of protein structures with atomistic detail can be beneficial for several pharmaceutical applications such as vaccine design^1^, drug discovery^2,3^, enzyme design^4^, self-assembling molecular machines^5^, and many more^6^. Mass spectrometry (MS) has become a prominent technique in the field of structural biology due to its ability to provide structural information for proteins and protein complexes. MS can be particularly beneficial because it is faster, can work for heterogeneous samples, can be used routinely at all stages of a project, and has fewer sample preparation complications compared to high-resolution techniques such as X-ray crystallography and cryo-electron microscopy (cryo-EM), and fewer concentration and size limitations compared to nuclear magnetic resonance (NMR) spectroscopy. Several findings for protein structures in the gas phase also suggest that features such as elements of secondary structure, compactness, and quaternary structure can be preserved during the transition from solution to desolvated state^7–9^. For these reasons, structural MS can be very beneficial particularly when high-resolution methods are not feasible^10,11^. Various methods have been developed and coupled to MS to study protein structures^12,13^ including in-solution approaches such as chemical crosslinking^14^, covalent labeling^15^ and hydrogen-deuterium exchange^16^, and gas-phase approaches such as collision-induced dissociation, electron capture/transfer dissociation, ultraviolet photodissociation, surface-induced dissociation^17^ and ion mobility (IM) spectrometry^18^. While such MS techniques may provide diverse details and routine analysis of structures, experimental data collected from experiments are sparse and cannot unambiguously determine atomic-resolution structure^19^.

An alternative approach to experimental structure determination is to use computational modelling methods. These approaches, such as structure prediction from sequence or protein-protein docking, can also provide insight into atomistic details of biomolecules but are frequently limited in accuracy due to the large conformational sampling space among other challenges^20^. While these methods can be successfully utilized in the absence of experimental data, sparse experimental data are often used to guide and improve modelling^19,21,22^. Experimental data from various MS techniques have recently proved pivotal within integrative structural biology frameworks^14,16,23–40^.

In IM, ions are transferred into an inert gas chamber at a constant pressure and temperature under the influence of a weak electric field^41,42^. This technique is regularly utilized to separate protein structures based on their shape and size. IM can also provide a rotationally averaged collision cross section (CCS_IM_) of the protein which is related to the amount of momentum exchanged between ion and buffer gas over the course of the collisions and can be thought of as somewhat like rotationally averaged cross sectional area^43^. Several methods have been developed to predict CCS from protein structure. Among these, the most physically realistic algorithms are the trajectory method (TJM)^44,45^ and diffuse trajectory method (DTM)^46^ which integrate Newton’s equation of motion to calculate the classical scattering of gas particles. Both TJM and DTM explicitly account for long-range interactions through Lennard-Jones potentials to approximate momentum transfer from each gas particle to the collided ion. CCS obtained from these methods is very accurate^45^, but these calculations can be slow. Due to the high computational cost, prediction methods such as elastic hard sphere scattering^47^, projection superposition approximation (PSA)^48^, local collision probability approximation^49^, and projection approximation (PA)^43^ make further approximations on TJM, resulting in faster CCS calculations. Among these approximated methods, PA is the simplest and fastest, because it neglects the scattering and long-range interactions^43,50^. CCS_PA_ only accounts for the collisions of a gas particle with the ion based on hard sphere atomic radii by calculating the average cross-sectional area of the protein structure as experienced by the buffer gas. Using the CCS projection approximation calculation tool IMPACT, calculations are about 10^6^ times faster^43^ than the most rigorous methods and are widely used for comparison with experimental IM data. Therefore, PA approaches are advantageous for use in integrative modelling, where the CCS calculation is required for thousands of structures that are obtained from Monte Carlo sampling.

Several instances of structural modelling in conjunction with IM data have been reported. IM spectra have been successfully predicted with the structure relaxation approximation (SRA) method^9^. This method uses molecular dynamics simulations to model structures in the specific charge states. It then utilizes CCS_PSA_ of the generated structures to predict an overall IM spectrum. The SRA method indicated that systems studied with IM methods are generally consistent with retention of many residue-residue contacts determined by X-ray crystallography. Several studies suggest that during native IM experiments, globular proteins undergo minimal compaction and structural rearrangement upon transfer to the gas phase using nanoelectrospray ionization, assuming they are appropriately charged (with lower charge states often exhibiting patterns consistent with solution structures) and that instrument conditions are kept “soft” to avoid unintentional activation^51–53^. Thus, the proteins largely retain native-like secondary, tertiary, and quaternary structures during the timescale of native IM experiments^9,40,54,55^. Therefore, data from such gas phase studies likely is beneficial for modelling solution phase structures though a rapid, validated way to evaluate plausible candidate structures consistent with IM-MS data would be beneficial to interpreting these results. Furthermore, IM data have been incorporated in computational modelling for protein complex structure prediction. In these methods, coarse-grained models generated using the Integrative Modelling Platform^56^ were ranked and clustered based on the agreement between predicted and experimental CCS measured from IM^28,35^. CCS_IM_ values for complexes and their individual subunits have also been successfully used to approximate rough intersubunit distance used as restraints in modelling methods to identify coarse-grained topologies of complexes^36–39^. Studies have also revealed that shapes and architectures of protein complexes can be determined from CCS_IM_ measurements and database searches^57,58^. In addition to complex structure prediction, work has also been done to show correlation between IM data and structural similarity (RMSD)^9^. While several studies have demonstrated that IM data can be predicted and utilized with various computational methods, IM data have not yet been utilized to predict monomeric structure from sequence.

Therefore, in this work a non-stochastic grid-based algorithm, PARCS, has been implemented in Rosetta^59,60^ to predict CCS from structure. It has been demonstrated that PARCS yields comparable results to IMPACT in terms of speed and accuracy. Next an IM score term has been developed for use in the ab initio^61–63^ and comparative modelling (CM)^64^ protocols in Rosetta, in combination with the Rosetta all-atom scoring function^65^. This score term scored structures based on their (dis)agreement with experimental IM data. When this score term was included, the prediction of structures improved for a benchmark of 25 proteins: the RMSD improved by an average of 2.0 Å and 17/25 structures were predicted accurately.

## Results

In this study, to utilize IM data to predict tertiary (monomeric) structures in Rosetta, an algorithm designed for rapid prediction of CCS has been developed and implemented. This method uses Projection Approximation (Eq. 1) via a grid-based calculation of Rough Circular Shapes (PARCS). Subsequently, a score function was developed (Eq. 2 and Eq. 3) that assessed the agreement of Rosetta-generated models to the CCS_IM_ for tertiary structure prediction.

### CCS Calculations by PARCS are fast, accurate and comparable to existing software

Area calculation in projection approximation methods is typically performed using Monte Carlo integration methods. In such an approach, probes representing the buffer gas particle are fired upon the randomly oriented 2D-projected target structure to calculate the area of the projection. A large number of probes is usually required for CCS calculations to converge. However, when a large number of probes is used, random probes frequently survey areas with no protein present, resulting in unnecessary calculations and thus adding to the computational cost^66^. Therefore, run-to-run variability in probe-based projection area calculation per rotation is common. To circumvent this issue, in PARCS, the projection area is calculated by projecting the structure on a 2D grid and then geometrically estimating the projection area directly (by geometrically filling the grid based on locations of atoms and radii of atoms and probes). This approach eliminates the variability in projection area calculation. Therefore, the only attribute contributing to the variability in CCS calculations using PARCS is the random rotations (Eq. 1).

**Fig. 1:**
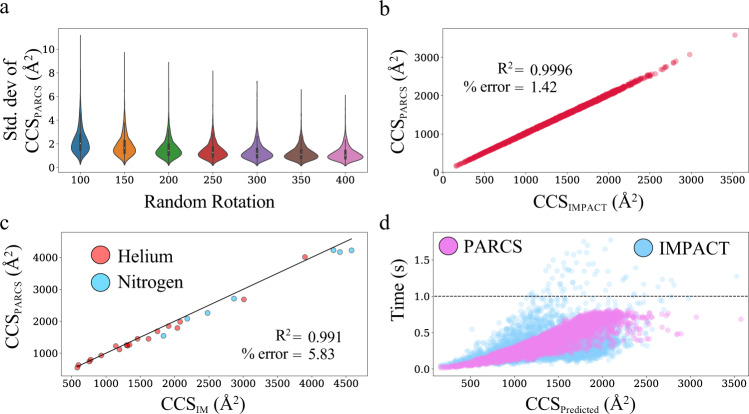
Analysis of projection area using rough circular shapes (PARCS) algorithm. **a** Convergence of collision cross section (CCS) calculation using PARCS was tested on, *n* = 4465, biologically independent samples (crystal structures deposited in the Protein Data Bank [PDB]) over 100 independent runs for 7 separate categories of random rotations ranging from 100R400 random rotations. The mean and the standard error of the mean for these distributions of 100R400 random rotations were 2.257 ± 0.016 Å^2^, 1.852 ± 0.013 Å^2^, 1.598 ± 0.011 Å^2^, 1.415 ± 0.010 Å^2^, 1.306 ± 0.009 Å^2^, 1.222 ± 0.009 Å^2^, 1.162 ± 0.008 Å^2^ respectively. The white dots represent the median in each violin distribution. The black bar in the center of the distribution is the interquartile range (IQR). The black stretched line extends from the “first quartile – 1.5 IQR” to the “third quartile + 1.5 IQR”. Values beyond this range are considered outliers. **b** Comparison of CCS_PARCS_ to that of CCS_IMPACT_ (of these *n* = 4465 biologically independent samples) exhibited a very strong correlation (*R*^2^ of 0.9996). **c** A strong correlation (*R*^2^ of 0.991) was observed for predicted CCS_PARCS_ values of PARCS when compared with CCS_IM_ from nitrogen (blue) and helium (red) buffer gas for the experimental dataset (*n* = 25 biologically independent samples with ion mobility data). **d** Comparison of CCS calculation time of PARCS and IMPACT (*n* = 4465 biologically independent samples) showed that PARCS and IMPACT performed equally well in terms of speed. Source data are provided as a Source Data file.

To benchmark our PARCS algorithm, CCS values for 4465 non-homologous protein structures in the PARCS evaluation dataset were calculated. Results for convergence of CCS calculations at varying number of random rotations on the PARCS evaluation dataset are shown in Fig. 1a. The average standard deviation of the CCS distributions for 100 rotations was only 2.26 Å^2^ (which was less than 0.2% of the CCS_PARCS_ on average) and decreased as the number of rotations increased. The average of the standard deviations of the CCS distributions was well below 2.0 Å^2^ for more than 100 rotations as shown in Fig. 1a. For CCS_PARCS_, the default number of rotations was set to 300, where the average standard deviation of the distribution was 1.31 Å^2^.

For all proteins in the PARCS evaluation dataset, CCS calculated by PARCS was compared to CCS calculated by IMPACT, one of the most widely used CCS calculation methods, as shown in Fig. 1b. A strong correlation (*R*^2^ = 0.9996) was observed between CCS_PARCS_ and CCS_IMPACT_ with a root mean squared error (RMSE) of 21.03 Å^2^ and an average percent error of 1.42%. The results demonstrate that PARCS calculates CCS values as accurately as other projection approximation methods. CCS_PARCS_ were then compared to CCS_IM_ for the experimental dataset. We observed a strong correlation (*R*^2^_PARCS_ = 0.991) between CCS_PARCS_ and CCS_IM_ values as shown in Fig. 1c, where IM data collected in nitrogen and helium buffer gas are shown in blue and red respectively. We observed an average percent error of only 5.83% (similar to that of IMPACT at 5.61%) from CCS_IM_. To use IM data in computational structure prediction methods (where CCS prediction is required on a large number of decoy structures), the speed of CCS calculations should be within about a second. Therefore, calculation times of PARCS were compared to that of IMPACT as presented in Fig. 1d. Using the PARCS evaluation dataset, PARCS took an average of 0.40 seconds to calculate the CCS of proteins when 300 random rotations were used compared to 0.32 seconds for IMPACT. Thus, the timing of PARCS was comparable to IMPACT. We note that the slightly longer average time for PARCS was due to additional steps performed by Rosetta when reading in a PDB file (such as checks for correctness and adding missing hydrogens^59^). For all 4465 proteins, calculations for PARCS completed in under 1.0 second as shown in Fig. 1d. These results indicate that PARCS in Rosetta offers similar speed and accuracy to established PA algorithms. We hypothesized that the information contained within CCS_IM_ may be sufficient to predict structures using CCS_PARCS_.

### PARCS in IM score function improves model selection in an ideal dataset

**Fig. 2:**
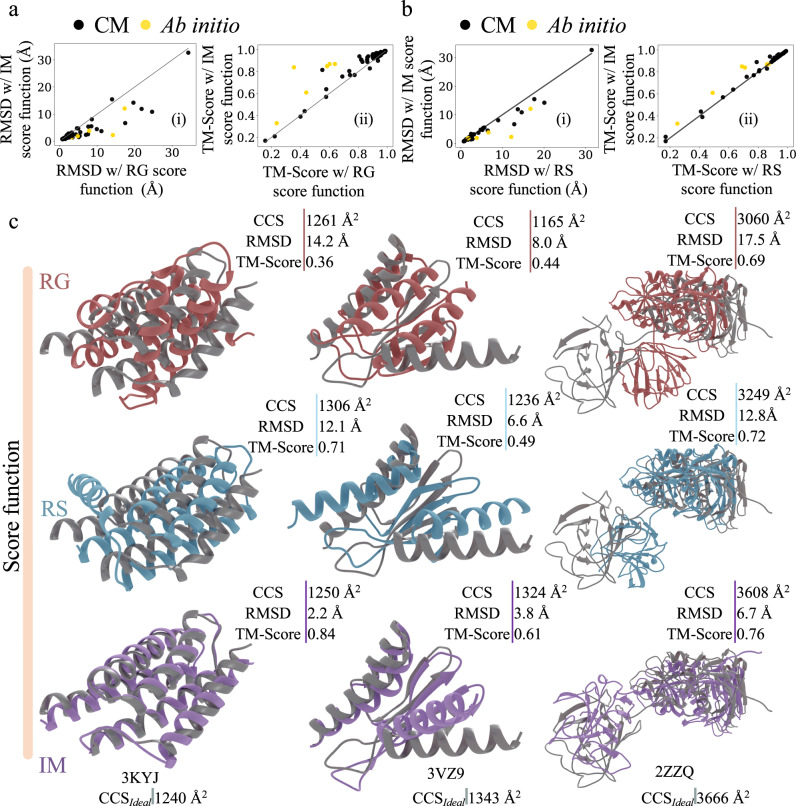
Protein structure prediction with and without ideal ion mobility (IM) data. Consistent improvement in model selection was observed when using the IM score function for the 60 proteins in the ideal dataset. The predicted models from the IM score function were compared to that of the **a** radius of gyration (RG) and **b** Rosetta (RS) score functions in terms of their respective (i) root mean square deviation (RMSD) and (ii) template modelling score (TM-Score). For both **a**, **b** models predicted with comparative modelling (CM) and ab initio are shown with black and yellow circles respectively. **c** Comparison of predicted structures with RG (pink), RS (blue) and IM (purple) score function to their native structures (grey) for three members of the ideal dataset. Source data are provided as a Source Data file.

In this study we sought to investigate the usefulness of the structural information encoded in IM data for predicting the complete structure of single-subunit proteins. However, it was unclear whether a single CCS value, encoding overall size and shape, was sufficient to distinguish near-native from incorrect protein models. To test how useful the information in CCS was for structure prediction, an IM score function (Eq. 2) has been developed to score structures based on the (dis)agreement with experimental IM data (Eq. 3). To assess the capabilities of this score function to adequately distinguish good from bad models, we first tested it on the ideal dataset (where the experimental CCS_IM_ was replaced with CCS_PARCS_ of the native structure for a set of proteins representative of all unique architectures in the CATH database, CCS_Ideal_). For each protein in the ideal dataset, 10,000 potential structures were generated (decoy structures), using the protocol outlined in Supplementary Methods (modelling protocol explicitly noted for each protein in Supplementary Data 1) and scored using the developed IM and RG score functions. Prediction results from the RG, RS, and IM score functions were evaluated and compared based on agreement with experimental structures (using the RMSD and TM-Score of the lowest scoring model, i.e., the predicted structure, Supplementary Data 2). We observed a significant improvement in model quality upon the inclusion of ideal IM data. The predicted structures with the IM score function were close to their native structures with an average RMSD of only 3.7 Å. The average TM-Score of these predicted models was 0.86. The models predicted with the RG score function (a proxy score function that only favors compact models) had an average RMSD and TM-Score of 5.7 Å and 0.80 respectively. As highlighted in Fig. 2a, the models predicted with the IM score function generally had lower RMSD (i) and higher TM-Score (ii) compared to those predicted by the RG score function. These results suggest that the two quantities (collision cross section and radius of gyration) do not provide the same structural information and that CCS-based scoring far outperforms modelling based solely on radius of gyration. Thus, RG cannot be used as a substitute for the IM score function. Structures predicted with the IM score function were then compared to those predicted with the RS score function (default Rosetta score function that did not utilize IM data). As shown in Fig. 2b (i), the RMSD of the predicted structures improved or remained unchanged for 58 out of 60 proteins, with an average RMSD improvement of 0.8 Å. The TM-Score also improved or remained unchanged for 58 proteins as shown in Fig. 2b (ii). The average TM-Score improvement over the RS score function was 0.02. Three of the predicted structures (that showed significant above-average improvement) from each the score function (RG, RS, and IM) are shown in Fig. 2c, where the native structure (grey) was compared to the predicted model of the RG (red), RS (blue), and IM (purple) score function.

The ability of the IM score function to predict structures as a function of sample size was also tested by varying the number of scored decoy structures from 100 to 10,000 for the ideal dataset. The average RMSD and TM-Score effectively improved for the IM score function as the number of predicted decoys increased (Supplementary Fig. 1a [i] and Supplementary Fig. 1a [ii] respectively). However, after about 7000 structures this improvement was negligible, suggesting that users should generate at least 7000 decoys.

Along with the improvement in model selection, the IM score exhibited a more well-defined energy funnel when compared to the RG and RS score functions. We saw a 9.5-fold increase in P_near_ when we used the IM score function over RS. We also observed a 22.4-fold increase in P_near_ with the IM over RG score function. This suggests that inclusion of IM data significantly improved the goodness of the score versus RMSD and TM-Score funnel. Finally, to test the robustness of the IM score function in the presence of experimental uncertainty, noise was introduced to the ideal CCS data (as outlined in Supplementary Methods). These “noisy” ideal CCS data were then used with the IM score function to predict structures. As shown in Supplementary Fig. 2a, the IM_score_ versus TM-Score distributions for all generated structures of the ideal dataset (600,000 structures) at 0% noise (blue, [i]), 15% noise (orange, [ii]), and 30% noise (green, [iii]) were largely identical. Furthermore, no significant change in the global fold was observed for the 60 best scoring models that were predicted with 15% and 30% random noise (Supplementary Fig. 2b). The average TM-Score of the predicted structures at 15% and 30% noise differed by only 0.01 and 0.02, respectively, when compared to that in the absence of noise for the ideal dataset. This suggests that the IM score function is not affected by reasonable amounts of random noise.

Due to the possibility of a gas-phase collapse, which would lead to a lower measured CCS than expected based on the protein crystal structures^67–70^, we also used the IM score function to predict structures based on artificially reduced CCS_Ideal_ values (for the ideal dataset) that were significantly lowered (up to 30%) compared to those found in the native structures (Supplementary Data 3). When these adjusted CCS values were used in scoring, the average radius of gyration (of the predicted models) decreased only slightly from 20.28 Å to 19.92 Å (as CCS_Ideal_ decreased by 30%) while the average radius of gyration of the native structures was 20.62 Å. This analysis indicated that even significantly smaller than expected CCS values induced only minimal compaction in the global fold of the predicted structures compared to the native models. This compaction is expected, since the IM data yields information about the shape and size of proteins, therefore lowering the CCS_Ideal_ will lead to prediction of models that are more compact. Several studies also indicate that minor gas-phase compaction could result due to the self-solvation of surface residue side chains^55,71–74^. Therefore, to test whether the observed minor compaction was simply due to the exposed side chains self-solvating in the gas phase, we also analyzed the neighbor count (NC) of surface residues (as defined earlier) in the predicted models using the same decreasing CCS_Ideal_ (Supplementary Data 3). A low NC generally corresponds to residues that are more solvent exposed and vice versa. Our results show that as CCS_Ideal_ was lowered (as described above), the IM models tended to exhibit surface residues that were only slightly less solvent exposed on average, suggesting that the side chains of these predicted models were only slightly collapsed compared to those of the native structures. However, the average difference of the surface residue NC (from that of the native structures) was only 0.78 when surface residues were defined as the residues with the lowest 20% of neighbor counts. Furthermore, the corresponding average RMSD increased to no more than 4.70 Å and the TM-Score decreased to no less than 0.84 when the CCS was artificially lowered up to 30%. These findings indicated that a small amount of side chain collapse for surface residues is the primary reason for the increase in compactness. Additionally, these results were expected since Rosetta used an implicit solvation model during model generation. As a result, gas-phase like structures were not likely to be found within the ensemble. Moreover, the IM_Score_Term_ was determined using an upper bound (UB) and lower bound (LB) as shown in Eq. 3. If the ΔCCS (absolute difference between the predicted and experimental CCS) was higher than the UB, then the structure in question was given a constant maximum penalty (Eq. 3). Effectively, this caused the ranking of structures using IM score function to be the same as the RS score function.

**Fig. 3:**
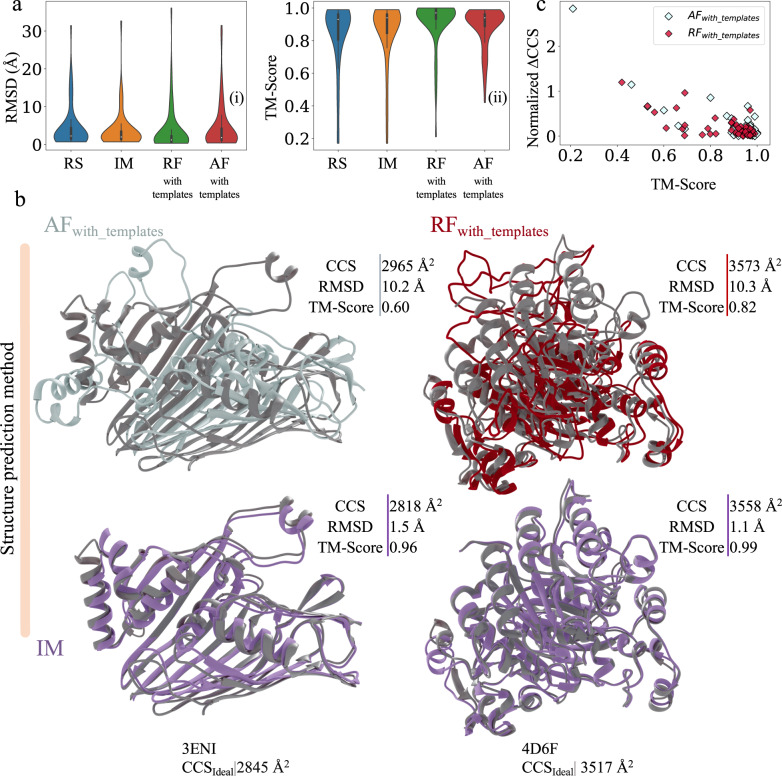
Comparison of predictions with ideal ion mobility (IM) data to those of AlphaFold (AF) and RF (RosettaFold). Predictions, for the ideal dataset, using the IM score function were compared to that of Rosetta (RS), AF_with_templates_, and RF_with_templates_. **a** Violin distributions (*n* = 60 biologically independent samples over 4 independent modelling approaches), of (i) root mean square deviation (RMSD) and (ii) template modelling score (TM-Score) of the predicted structure using the RS, IM, AF_with_templates_ and RF_with_templates_ score functions. For protein structure predictions with methods shown in **a** the mean and the standard error of mean in (i) are 4.46 ± 0.74 Å, 3.72 ± 0.65 Å, 3.43 ± 0.77 Å, and 4.37 ± 0.80 Å respectively. Similarly the mean and the standard error of mean in (ii) are 0.84 ± 0.03, 0.86 ± 0.02, 0.92 ± 0.02, and 0.88 ± 0.02 respectively. For the violin distributions in a (i) and a (ii) the white dots represent the median. The black bar in the center of the distribution is the interquartile range (IQR). The black stretched line extends from the “first quartile −1.5 IQR” to the “third quartile + 1.5 IQR”. Values beyond this range are considered outliers. **b** Comparison of predicted structures with AF_with_templates_ (cyan), RF_with_templates_ (red) and IM (purple) to their native structures (grey) for the ideal dataset. **c** High normalized absolute difference in collision cross section of the predicted structure and the native structure (ΔCCS divided by sequence length) for structures predicted with AF_with_templates_ (cyan) and RF_with_templates_ (red) generally corresponded to structures with low TM-Score as seen for the ideal dataset. Source data are provided as a Source Data file.

Protein structures in the ideal dataset (60 proteins) were also predicted with AlphaFold2 (AF) and RoseTTAFold (RF). These structures were predicted both with and without templates (as outlined in Supplementary Methods). Furthermore, we benchmarked the prediction results of AF and RF both with and without templates for the ideal dataset as shown in Supplementary Fig. 3. For the ideal dataset, for both AF and RF, the average RMSD decreased by 0.2 Å and 0.33 Å respectively when templates were used. Similarly, the average TM-Score increased by 0.01 and 0.04 when templates were used for predicting the structures of proteins. In Fig. 3a, all three methods (IM, AF_with_templates_, and RF_with_templates_) predicted better structures than RS (Supplementary Data 2). For the ideal dataset, the average RMSD of the predicted models from the IM score function was higher than those predicted with AF_with_templates_ by 0.3 Å. In contrast, the average RMSD of IM predicted models were lower by 0.6 Å when compared to those predicted with RF_with_templates_. These results are highlighted in Fig. 3a (i). The average TM-Score of the IM predicted models were lower than AF_with_templates_ and RF_with_templates_ by 0.06 and 0.03 respectively as shown in Fig. 3a (ii). However, there were 28 cases where the IM predicted models that were better or the same when compared to AF_with_templates_ models. The average RMSD and TM-Score for this subset improved by 2.7 Å and 0.04, respectively. IM also predicted 37 models that were better or the same when compared to RF_with_templates_ models. For this subset, the average RMSD and TM-Score improved by 2.6 Å and 0.05 respectively. Figure 3b shows two such cases where the predicted structure using IM (purple) matches the native (grey) significantly more closely over the predicted AF_with_templates_ (cyan) and RF_with_templates_ (red) structures. Additionally, the average absolute percent error of CCS_PARCS_ to CCS_Ideal_ of the top scoring models of RG, RS, AF_with_templates_, RF_with_templates_, and IM were 4.6%, 3.1%, 2.4%, 2.2% and 0.9% (Supplementary Data 4). This is expected because the IM score function predicts models that agree the most with IM data as compared to all other methods that do not use this information for structure prediction. Furthermore, large deviations in normalized ΔCCS (ΔCCS divided by sequence length) were also observed for poorly predicted AF_with_templates_ and RF_with_templates_ structures (TM-Score below 0.5) as shown in Fig. 3c. Similar results were also observed for poorly predicted AF_without_templates_ and RF_without_templates_ structures as shown in Supplementary Fig. 4 (and Supplementary Data 5).This suggested that in the future the IM score function could also be used to assess structures generated with AF and RF (both with and without templates). Given the sparseness of the data (CCS is a single number denoting the average cross-sectional area of the protein) these results indicated that the overall size and shape information contained in the IM data indeed had a strong potential to facilitate the discrimination of good from bad models. While an encouraging proof of principle, these results do not account for the uncertainty associated with real experimental IM data. An average percent error of 5.83% between CCS_PARCS_ and CCS_IM_ was observed for the experimental dataset (Fig. 1c). Thus, when we turn to experimental IM data for the structure prediction, additional uncertainty will be present. Therefore, we next present tests of the effectiveness of IM data to improve structure prediction that are based on use of a dataset with experimental IM data.

### IM data improve model selection of protein structures in an experimental dataset

**Fig. 4:**
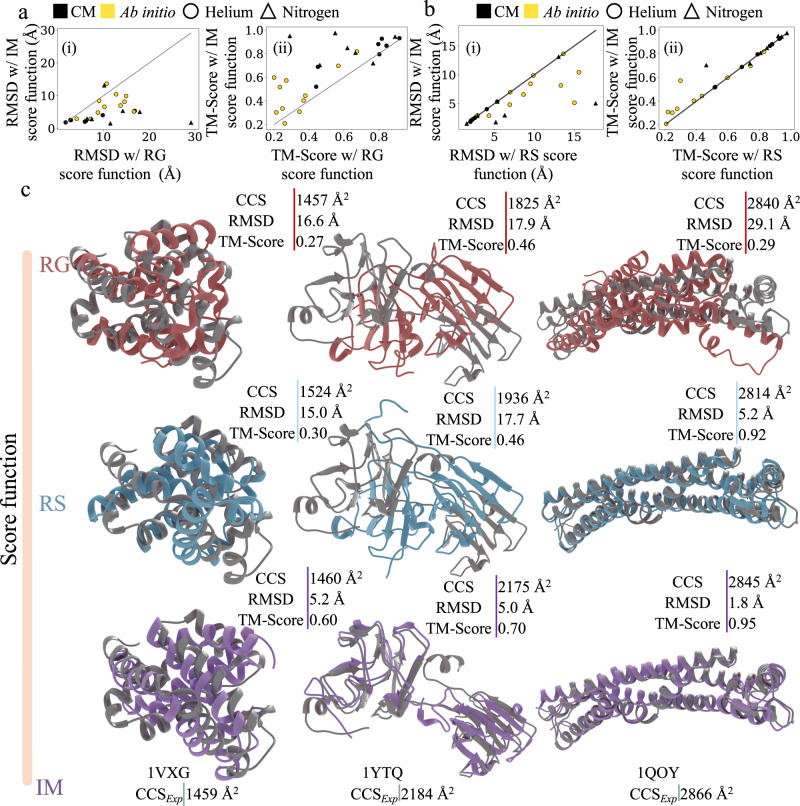
Protein structure prediction with and without experimental ion mobility (IM data). Consistent improvement in model selection using the IM score function was observed for the 25 proteins with experimental IM data. The predicted models from the IM score function were compared to those of the **a** radius of gyration (RG) and **b** Rosetta (RS) score functions in terms of their respective (i) root mean square deviation (RMSD) and (ii) template modelling score (TM-Score). For both **a**, **b**, circle and triangle indicate IM data collected in helium and nitrogen buffer gas respectively; while models predicted with comparative modelling (CM) and ab initio are shown in black and yellow respectively. **c** Comparison of predicted structures using the RG (pink), RS (blue) and IM (purple) score function to their native structures (grey). Source data are provided as a Source Data file.

**Fig. 5:**
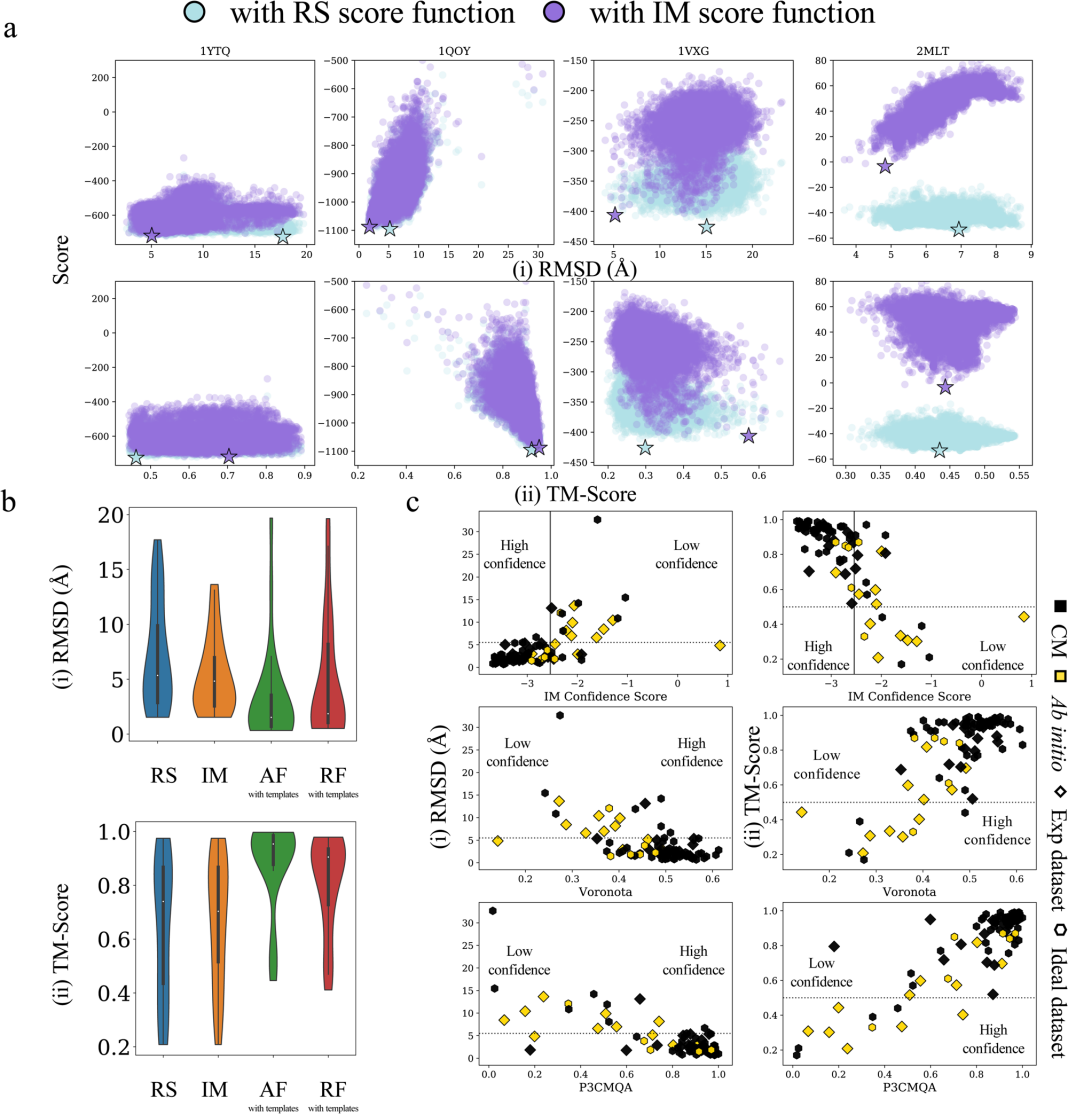
Protein structure prediction results for the experimental dataset and model quality assessment. The score distribution improves to a more funnel-like shape with the ion mobility (IM) score function for the experimental dataset. **a** This is highlighted by the score vs (i) root mean square deviation (RMSD) and (ii) template modelling score (TM-Score) distributions of four proteins that showed significant improvement with the IM (purple) score function over Rosetta (RS, blue) score function. The best scoring models from both predictions are marked with a blue and purple star for RS and IM, respectively. **b** Violin distributions, *n* = 25, biologically independent samples over 4 independent modelling approaches of (i) RMSD and (ii) TM-Score of the predicted structures with RS, IM, AlphaFold with templates (AF_with_templates_) and RosettaFold with templates (RF_with_templates_). For protein structure predictions with methods shown in **a** the mean and the standard error of mean in (i) are 7.18 ± 0.99 Å, 5.33 ± 0.70 Å, 3.13 ± 0.90 Å, and 4.57 ± 1.08 Å respectively. Similarly, the mean and the standard error of mean in (ii) are 0.64 ± 0.05 Å, 0.67 ± 0.05 Å, 0.88 ± 0.03 Å, and 0.81 ± 0.04 Å respectively. For the violin distributions in **b** (i) and **b** (ii) the white dots represent the median. The black bar in the center of the distribution is the interquartile range (IQR). The black stretched line extends from the “first quartile – 1.5 IQR” to the “third quartile + 1.5 IQR”. Values beyond this range are considered outliers. **c** Comparison of (i) RMSD and (ii) TM-Score of the predicted structures (with IM) from both the experimental (diamond) and ideal (hexagon) dataset vs IM confidence score. Models generated with comparative modelling (CM) and ab initio are shown in black and yellow respectively. Similarly, the Voronota and P3CMQA scores are shown. Source data are provided as a Source Data file.

For proteins in the experimental dataset, 10,000 decoy models were generated with either the ab initio or comparative modelling (CM) protocols as specified in Supplementary Data 6. Each of these decoy models was scored with IM data (Eq. 2) and the predicted models were then compared to those scored with the RS and RG score functions (Supplementary Data 7). Again, we saw a notable improvement in model quality upon the inclusion of IM data. In Fig. 4a and b, (i) the RMSDs (and [ii] TM-Score) of the best scoring models with IM data are compared to those from the RG and RS score functions. As highlighted in Fig. 4a (i), the average RMSD of the predicted structures with the IM score function was 5.3 Å, while the average RMSD for those predicted with the RG score function was 10.6 Å. Similarly, the average TM-Score of the IM and RG predicted structures were 0.67 and 0.52 respectively (Fig. 4a [ii]). These results further established that model discrimination using actual experimental IM data significantly outperforms the simple proxy score function (RG) that only ensured protein compactness. Compared to the RS score function as shown in Fig. 4b (i), the RMSDs of the predicted models for proteins in the experimental dataset either improved or remained unchanged in all 25 cases. Similarly, the TM-Score either improved or remained unchanged for 22/25 proteins (Fig. 4b [ii]). The RMSD improved by an average of 2.0 Å (average TM-Score improvement of 0.03) when IM data were utilized as restraints. Of these 25 cases, 17 proteins were ultimately predicted with an RMSD of less than 5.5 Å, compared to 13 proteins without IM data (RS score function). Figure 4c shows structures of the predicted models (aligned to the native structures in grey) obtained with the RG (pink), RS (blue), and IM (purple) score functions. The largest RMSD improvement was observed for the system β-crystallin B2 (PDB ID: 1YTQ), whose RMSD decreased from 17.7 Å to 5.0 Å. The TM-Score for this protein improved from 0.46 to 0.70 when the predicted structure from RS was compared to that of the IM score function. Similarly, for the system Hemolysin E (PDB ID: 1QOY), the IM score function predicted a significantly better model when compared to that predicted by the RG score function. The RMSD and TM-Score differences (between the models selected by the IM and RG score functions) were 27.3 Å and 0.66 respectively. No significant difference was observed in IM score model selection with respect to IM data collected from the two different experimental conditions (helium and nitrogen buffer gas). This suggests that IM data from both buffer gases are equally useful for modelling. The score vs RMSD and TM-Score distributions for several benchmark proteins using the RS (blue) and IM (purple) score functions are shown in Fig. 5a (i) and Fig. 5a (ii) respectively. In these distributions, the predicted models from RS and IM are marked with a blue and purple star, respectively. We observed a general improvement in P_near_ upon scoring with the IM score function with a 4.6-fold average improvement over that of the RS score function (Supplementary Data 7). The average P_near_ also increased by 43.4-fold when the score distributions were compared to those from the RG score function. This showed that the goodness of the score vs RMSD and TM-Score distribution is generally improved when IM data are included to predict structures. For comparison purposes, protein structures were also predicted with AF_with_templates_ and RF_with_templates_ for the experimental dataset as shown in Fig. 5b. The predicted models were compared to their native structures by RMSD as shown in Fig. 5b (i) (and Fig. 5b [ii] for TM-Score). On average, AF_with_templates_ and RF_with_templates_ both predicted structures better than the IM score function. Additionally for the experimental dataset, we observed that use of templates with AF and RF improved the RMSD and TM-Score difference when compared to prediction results without templates (Supplementary Fig. 5). The RMSD difference improved by 0.58 Å and 1.94 Å for AF and RF respectively. Similarly, the TM-Score difference for AF and RF improved by 0.03 and 0.04 respectively. Similarly, structures predicted with the IM score function were better than those predicted with RS (without the aid of IM data). Despite the impressive performance of AF and RF, there were several cases where inclusion of IM data outperformed those predictions. There were 6 cases (out of 25 proteins in the experimental dataset) where structure prediction was better or the same, with the IM score function when compared to that of AF_with_templates_ (Supplementary Data 7). The average RMSD and TM-Score difference of this subset were 3.2 Å and 0.04 respectively. Similarly, there were 9 cases (Supplementary Data 7) where predictions from IM outperformed those models predicted by RF_with_templates_. The average RMSD and TM-Score of this subset differed by 5.4 Å and 0.13. Furthermore, the average absolute percent error of CCS_PARCS_ to CCS_IM_ of the top scoring models of RG, RS, RF_with_templates_, AF_with_templates_, and IM were 8.6%, 7.6%, 6.0%, 5.5%, and 5.4%, respectively (Supplementary Data 8). This confirmed that the IM score function predicts models that best agree with the experimental IM data when compared to all other structure prediction methods (RG, RS, RF, and AF). Furthermore, we focused on a subset of 54 proteins from both the experimental and ideal dataset for which either the CM protocol with non-perfect templates or the ab initio protocol (template-free modelling) were performed. For this subset, we defined non-perfect templates as templates with sequence identity and coverage (to target protein) ranging anywhere from 14% to 84% and 6% to 100% respectively. Next, for this subset we compared the best scoring models to their native structures for the radius of gyration (RG), Rosetta (RS), and ion mobility (IM) score functions. Again, we observed consistent improvement in model selection for the IM score function over the RG and RS score functions, for both the ideal and experimental dataset as shown in Supplementary Fig. 6. Compared to the RG score function, the IM score improved the RMSD of the selected model (for all the 54 proteins in this subset, Supplementary Data 9) by 2.72 Å on average. The TM-Score for this comparison also improved by 0.08 on average. Similarly, compared to the RS score function, the IM score improved the RMSD of the selected model by 1.12 Å and the TM-Score by 0.02. A summary of this analysis can be found in Supplementary Data 10. Thus, we conclude that IM data can improve protein structure prediction both in complete absence of templates and in the presence of non-perfect templates. In summary, these results demonstrated that experimental IM data can offer shape and size information that can be used to improve protein structure prediction.

### IM confidence score discriminates accurate and inaccurate models

The inclusion of IM data helped improve structure prediction for all 25 proteins in the experimental dataset. However, there were 8 cases where the RMSD of the selected model (even after improvement) was greater than 5.5 Å (five of those eight cases had TM-Scores less than 0.5). This knowledge was available to us since the native structures were known for the models generated within this benchmark dataset. However, in true blind structure prediction protocols, RMSD or TM-Score information is not available. For this reason, we developed a confidence measure, the IM confidence score, that allowed us to selectively flag successful prediction cases in the absence of native structure. The IM confidence score was defined as the average score of the top 100 scoring models divided by sequence length. According to this metric analysis, the high and low confidence structures were separated by a score cutoff of −2.54. This metric flagged all inaccurate predictions as low confidence, whereas all high confidence predictions were accurate. We tested whether the IM confidence score made predictions that surpassed or at least matched other software that can assess model quality in the absence of IM data. For this purpose, IM confidence score results were benchmarked against two other model quality assessment programs (Voronota and P3CMQA) as shown in Fig. 5c for both the experimental (diamond) and ideal dataset (hexagon). To better compare the performance of the IM confidence score to Voronota and P3CMQA, the confidence score was scaled (as outlined in Supplementary Methods) such that it ranged from 0 to 1, with 1 being the most confident model and 0 being the least confident (similar to the convention in Voronota and P3CMQA) as shown in Supplementary Fig. 1 (b) with (i) RMSD and (ii) TM-Score. Our results (Fig. 5c and Supplementary Fig. 1 [b]) indicated that the IM confidence score were comparable to that of Voronota and P3CMQA.

## Discussion

Ion mobility (IM) has emerged as a prime tool to study proteins in their native states using mass spectrometry (MS) due its ability to conserve native-like structural information in the gas phase. Furthermore, native IM-MS measurements are relatively fast, use very little sample, and are highly chemically specific, making them both relatively easy and informative as compared to many other types of structural biology techniques. Additionally, native IM-MS measurements are not limited by the size of the system. For these reasons, IM-MS provides a wealth of structural information and can be used as routine analysis when compared to many other types of experimental techniques. However, the information obtained is sparse, not directly allowing for full structure elucidation. Thus, computational techniques are needed to deduce structural information from IM data. In this study we developed a new algorithm for structure prediction of single subunit proteins from IM data. To achieve this, we first developed a method (PARCS) that could predict collision cross section (CCS) from structures, which has been implemented in Rosetta as a stand-alone application. Following the successful benchmarking of this application, a score term, based on restraints derived from IM data, has been developed to predict native-like structures. This score term was tested on a set of 60 structures from the PDB, where CCS_PARCS_ (with simulated noise) of the native structure was treated as the experimental CCS_IM_. This was done, as a proof of principle, to check whether the score function could translate the structural information (encoded in IM data) into spatial restraints in the absence of model error. Based on RMSD and TM-Score analysis, we observed that the inclusion of IM data (to the Rosetta score function) improved structure prediction results for 58 out of 60 structures. Since radius of gyration (R_g_) has previously been used as a simple substitute for IM data, we tested the IM score function against a proxy score function that utilized R_g_ to favor compact models. From our results, we conclude that the two quantities (CCS and R_g_) point to different structures and thus a simple compactness-based proxy score function cannot be used as a substitute for the IM score function. Following this positive validation, the score function was tested on a benchmark set of 25 proteins with experimental IM data. We showed that IM data improved model selection, as demonstrated by analyzing the best scoring models with several metrics. Next, we benchmarked our method against recently developed structure prediction methods, AlphaFold2 (AF), and RoseTTAFold (RF), for both the ideal and experimental datasets. Despite the remarkable accuracy of these deep learning methods, our results show that there were several cases where the IM score function could improve structure prediction over AF and RF. Furthermore, our results also suggest that large deviations of predicted CCS from experimental CCS for AF and RF structures are indicative of models with poor fold, further underscoring the usefulness of IM data at aiding accurate structure identifications. We also developed a confidence metric (IM confidence score) to successfully separate good predictions from bad predictions in the absence of native structure. Our current computational workflow illustrates that CCS obtained from IM experiments, despite its sparseness, provides sufficient information on the overall shape and size of proteins to be used as restraints to improve model selection in protein structure prediction. Furthermore, our results also suggest that, despite its extensive assumptions and approximations, the projection approximation method is sufficient in an integrative modelling pipeline and provides the ability to rapidly compare large numbers of computed results to experiment, which can be very time-consuming with more physically explicit methods such as the Trajectory Method. With these benchmarks in place, future investigation using these higher-level CCS computation methods could help refine the model further. This study also demonstrates how information from native IM techniques (gas phase) can be used to successfully infer solution structure. Our developed CCS calculation method and score function are freely and easily accessible through Rosetta Commons. Supplementary Note 1 shows examples on how to use the PARCS application and Supplementary Note 2 contains instructions on the use of IM data in structure prediction. Furthermore, all related data (including all models generated) can be found in our GitHub repository^75^ (10.5281/zenodo.6726418). Further work will focus on improving methods to incorporate CCS data for protein complexes using RosettaDock^76^ and on the use of multiple complementary data types (such as the combination of covalent labeling^77–79^, surface-induced dissociation^80^, cryo-EM^81,82^ and/or NMR^83,84^ with IM data) for protein and protein complex structure prediction in Rosetta.

## Methods

### Projection approximation using rough circular shapes

**Fig. 6:**
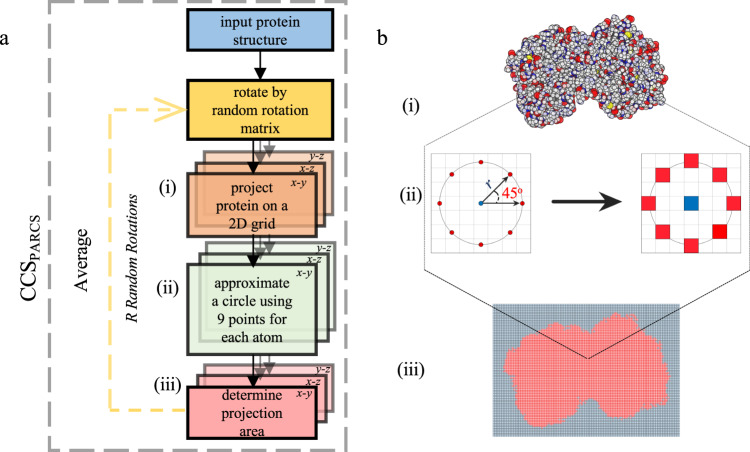
Overview of collision cross section (CCS) calculation using rough circular shapes (PARCS) algorithm. **a** Schematic of the PARCS algorithm to predict CCS from structure. (i) Three projections are obtained from each rotation. (ii) For each atom in each projection the 2 dimensional (2D) grid is filled according to a 9-point circle approximation. (iii) The projection area is determined from the number of filled grid cells. **b** Illustration of a (i) 2D projection of a single random rotation where the carbon, sulfur, oxygen, nitrogen, and hydrogen are colored grey, yellow, red, blue, and white respectively. (ii) Each atom is projected on a grid with a cell size of 1 Å^2^. The center grid cell and eight other grid cells at a distance *r* (based on the radii of the given atom and the buffer gas) from the center of the atom are filled. (iii) Projection of the randomly rotated protein after the grid cells are filled according to the PARCS algorithm.

Average CCS of biomolecules are determined from IM experiments based on the amount of time required for the ion to traverse the region of inert buffer gas (usually helium or nitrogen) under the influence of a weak electric field^43,45^. To use IM data in a structure prediction protocol, we developed Projection Approximation using Rough Circular Shapes (PARCS) in Rosetta. The schematic (a) and the illustration (b) in Fig. 6 demonstrate how the PARCS algorithm computes CCS from structure and estimates area of a projection, respectively. The PARCS algorithm, as shown in Fig. 6a, takes 3D atomic protein coordinates as input. Next, the structure is randomly rotated. For each rotation, the structure is projected on a 2D grid (grid cell area of 1 Å^2^) in the *x-y*, *x-z*, and *y-z* planes as shown in Fig. 6a (i). In the 2D grid, the projection of the protein is centered, and the grid extends 5 Å beyond the most extreme atom in each direction. For each atom on the 2D grid (Fig. 6a [ii]), the center grid cell is filled as denoted by the blue grid cell in Fig. 6b (ii). Then, eight additional cells (red grid cells in Fig. 6b [ii]) are also filled. The distance of these eight grid cells from the central cell (i.e., radius of the circular projection) is based on the sum of the radii of the projected atom and the buffer gas (*r* in Fig. 6b [ii]). An effective atomic cross-sectional radius of 1.91 Å is used for heavy atoms (carbon, sulfur, oxygen, nitrogen, and phosphorous) and 1.21 Å is used for hydrogen atoms. A buffer gas radius of 1.0 Å and 1.82 Å is used in the case of helium^43^ and nitrogen^85^, respectively. The eight points are positioned such that two adjacent points on the circumference form a 45^o^ angle from the center point as shown in Fig. 6b (ii). This process is repeated for all atoms in the protein, filling the overall grid as shown in Fig. 6b (iii). Finally, the projection area ($A$) is derived by summing the areas of the filled grid cells. From the *x-y*, *y-z*, and *x-z* projections for each random rotation, three projection areas (*A*_*i*_^*x-y*^, *A*_*i*_^*x-z*^ and *A*_*i*_^*y-z*^) are obtained. The CCS of the structure (CCS_PARCS_) is then acquired from the average area of the total number of projections (*N* = 3 *R*, where *R* is the total number of random rotations) as shown in Eq. 1.

1 $${{{{{{\rm{CCS}}}}}}}_{{{{{{\rm{PARCS}}}}}}}=\tfrac{\mathop{\sum }\nolimits_{i}^{R}\left({A}_{i}^{x-y}+{A}_{i}^{x-z}+{A}_{i}^{y-z}\right)}{N}$$

### IM score function in Rosetta

CCS from experimental IM data were incorporated as a spatial restraint for integrative Rosetta modelling as it provides information about protein size and shape. Therefore, to integrate this information in Rosetta for protein structure prediction, a score term (IM_Score_Term_) was developed to quantify agreement of protein structures with IM data, using CCS as the restraint. The evaluation score, IM_Score_, was defined as a sum of the IM_Score_Term_ score term with the Rosetta REF2015 score function^65^ as shown in Eq. 2.

2 $${{{{{{\rm{IM}}}}}}}_{{{{{{\rm{Score}}}}}}}={{{{{\rm{RS}}}}}}+{{{{{{\rm{IM}}}}}}}_{{{{{{\rm{Score}}}}}}\_{{{{{\rm{Term}}}}}}}$$

In Eq. 2, RS is the energy of the structure obtained from the Rosetta REF2015 score function. The IM_Score_Term_ term is a penalty function (as defined and shown in Eq. 3 and Supplementary Fig. 7, respectively) based on the absolute difference (ΔCCS) between CCS_PARCS_ and CCS_IM_. This function includes a lower bound (LB) and an upper bound (UB) cutoff (as shown in Eq. 3) to account for error^24^. ΔCCS below LB (10 Å^2^) are not penalized and ΔCCS above UB (100 Å^2^) are given a maximum penalty of 100, with a fade function used in between. Conceptually, this scoring function penalizes structures with high deviation from experiment.

3 $$\begin{array}{c}{{{{{{\rm{IM}}}}}}}_{{Scor}e{{{{{\rm{\_}}}}}}{Term}}=\begin{array}{c}\left\{\begin{array}{cc}0 & {if}\Delta {{{{{\rm{CCS}}}}}} < {{{{{\rm{LB}}}}}}\\ 100\left(2{x}^{3}-3{x}^{2}+1\right) & {if}\,{{{{{\rm{LB}}}}}} < \Delta {{{{{\rm{CCS}}}}}} < {{{{{\rm{UB}}}}}}\\ 100 & {if}\,\Delta {{{{{\rm{CCS}}}}}} > {{{{{\rm{UB}}}}}}\end{array}\right.\end{array}\\ x=-\left(\frac{\Delta {{{{{\rm{CCS}}}}}}-{{{{{\rm{UB}}}}}}}{{{{{{\rm{UB}}}}}}-{{{{{\rm{LB}}}}}}}\right)\end{array}$$

### IM datasets

In this work, our aim was to study predominantly globular and ordered proteins within all datasets. Values from CCS_PARCS_ were compared to CCS_IMPACT_ as well as evaluated for speed and precision on 4465 non-homologous protein structures (PARCS evaluation dataset) extracted from the protein databank (PDB)^86^ (http://www.rcsb.org/) using the PISCES^87^ webserver (http://dunbrack.fccc.edu/pisces). For this dataset (all PDB IDs used can be found in the Source Data file), the sequence identity was less than or equal to 10%, sequence length was between 40–250 residues, non-X-ray and CA-only entries were excluded and the PDBs were culled by chain. For CCS prediction and speed comparison, PARCS was benchmarked against IMPACT^43^ (with flag ‘-H’ to include hydrogens) based on the calculations performed on the PARCS evaluation dataset. This dataset was also used to test the convergence of PARCS with respect to the number of rotations. In this convergence test, the standard deviation of 100 separate CCS calculations for each protein at varying numbers of rotations were obtained and assessed for the optimal number of random rotations required for calculations to converge.

To evaluate the ability of the score term (Eq. 3) to distinguish native from non-native protein models in the case of an error-free CCS prediction, a set of 60 proteins was selected from the PDB such that it contained all unique architectures (list of monomers shown in Supplementary Data 1) as classified by the CATH Protein Structure Classification database^88^. The sequence length for proteins in this dataset ranged from 58 to 965. A set of structure prediction experiments (which will be described in detail in the following sections) was performed on this dataset, where the experimental CCS was simulated by predicting CCS of the native structure with PARCS. Therefore, this dataset was referred to as the ideal dataset. The simulated CCS (CCS_Ideal_) values ranged from 767 Å^2^ to 4130 Å^2^ for the 60 proteins in the ideal dataset. Furthermore, to address the effect of uncertainty in CCS values when using the IM score function (Eq. 2), varying degrees of noise were introduced to the simulated CCS data (as outlined in Supplementary Methods). The score function was also tested on actual experimental IM data, i.e., structures with CCS_IM_ (experimental dataset). The experimental dataset^18,89–93^ consisted of 25 monomeric proteins that also had structural information deposited in the PDB (as outlined in Supplementary Data 6). Sequence lengths ranged from 26–691 residues and CCS_IM_ values (for the lowest charge states) ranged from 588 Å^2^ to 4580 Å^2^. Additionally, the proteins exhibited an average percent disorder of only 13.2% and 10.7% as calculated by the Rosetta ResidueDisorder^94,95^ application for the experimental and ideal dataset respectively.

### Ab initio, comparative modelling, AlphaFold2, and RoseTTAFold protocols for structure prediction

To test whether shape and size information encoded in IM data were sufficient to discriminate between low and high RMSD (and TM-Score) models of single-subunit proteins, we tested our algorithm on both the ideal and experimental dataset. For these two datasets, the Rosetta (v.3.1.3) ab initio protocol was used for proteins with sequence length less than 155 residues, otherwise the Rosetta multi-template comparative modelling (CM) protocol was used. The templates and weights associated with all proteins for CM are provided in Supplementary Data 11 and Supplementary Data 12 for the ideal and experimental dataset respectively. The 3mer and 9mer fragments required for both protocols were generated using the fragment picker tool^96^ in Rosetta. The protocols (ab initio and CM) for both the ideal and experimental data set are further detailed in the Supplementary Methods. All structures generated from the ab initio and comparative modelling protocols (including the recovered structures for selected proteins in the experimental dataset as shown in Supplementary Data 13) were subjected to the Rosetta Relax protocol (term referred to as RS in Eq. 2). The IM data, ideal and experimental, were then used to score all the structures generated for each protein in Supplementary Data 1 and Supplementary Data 6, respectively. Radius of gyration has previously been used as proxy for IM data^97^. Therefore, the IM score function was also benchmarked against a simple proxy score function (RG) that was solely based on radius of gyration (described in the Supplementary Methods section), and thus favored compact models for proteins in both datasets. The top scoring model was designated as the predicted structure. AlphaFold2 (v.2.0.0)^98^ and RoseTTAFold (v.1.1.0)^99^ (protocols detailed in the Supplementary Methods) were also benchmarked (with and without templates) for both the datasets to further assess the effectiveness of IM data.

### Analysis metrics used for evaluating predictions

We quantitatively assessed the quality of our predicted models using several of the following metrics. The global RMSDs (root-mean-square deviations) of the predicted models to their native structures were calculated. Predictions with IM data where RMSD was within 0.5 Å of the RMSD of the structure predicted without IM data were defined as unchanged. Next, P_near_^100^, a goodness-of-energy funnel metric (at *k*_*B*_*T* and *λ* set to 10 and 1 Å respectively), was used to compare the score versus RMSD distributions predicted with the RG, RS, and IM score functions. P_near_ ranges from 0 (a poor energy funnel) to 1 (a well-defined energy funnel). All predicted structures from both ideal and experimental datasets were further evaluated with the template modelling score (TM-Score)^101^. TM-Score was used to assess the topological similarity of the predicted structures to native structures using the TM-Score program^101^. The TM-Score metric ranges from 0 to 1, where scores below 0.17 indicate randomly chosen unrelated proteins and a score higher than 0.5 corresponds to structures being generally in the same fold and a score of 1 indicates a perfect match^101^.

### Confidence metric used for identifying accurate and inaccurate predictions

A metric was developed (IM confidence score) to quantify confidence in predictions in the absence of known structure. The IM confidence score was defined as the average score of the top 100 scoring models predicted with IM data (using Eq. 2) divided by the sequence length (i.e., average score per residue). The specific metric was chosen because lower scores per residue are generally associated with more native-like structures. Thus, structures were defined as high confidence if the average residue score was less than −2.54 (above which structures were defined as low confidence). Instances where the RMSD of the prediction was less than 5.5 Å (correspondingly above 0.5 TM-Score) and the average residue score was less than −2.54 were considered successful confidence measure cases. We chose an RMSD cutoff of 5.5 Å (TM-Score cutoff of 0.5) since below that RMSD (and above that TM-Score), protein topologies are generally predicted correctly. The IM confidence score was further tested against two other model quality programs Voronota (v1.22.3149)^102^ and P3CMQA (v.1.0.0)^103^ (protocol outlined in Supplementary Methods).

### Analysis of compactness of model selected with IM score function

We also investigated (using the ideal dataset) whether models predicted with the IM score function were biased towards a possible gas phase energy minimum which might show signs of structural compaction. To do this, we reduced the CCS_Ideal_ by 2% to 30% and rescored with the IM score function. We subsequently analyzed the top scoring structures (using the reduced CCS_Ideal_ as part of scoring) for compactness using radius of gyration. Next, we calculated the neighbor count (NC) of all residues with the Rosetta application per_residue_solvent_exposure^25^ (using the sphere method with default parameters). A residue with high NC is approximated as buried, while a residue with low NC is thought to be solvent exposed. Therefore, we analyzed the NC of surface residues at varying reduced CCS_Ideal_. The surface residues were approximated as the top X % residues with the lowest NC in the native structure, where X varied from 5% to 20%.

### Software usage for data analysis

Python v.3.7.3 was used for data analysis. Matplotlib v.3.1.2 was used for the creation of all scatter plots, line plots and violin distributions. PyMOL v.2.0.6 and Blender v.2.8.1 were used in combination to generate the figures of all proteins.

### Reporting summary

Further information on research design is available in the Nature Research Reporting Summary linked to this article.

## Supplementary information

Supplementary Information

Description of Additional Supplementary Files

Supplementary Dataset 1

Supplementary Dataset 2

Supplementary Dataset 3

Supplementary Dataset 4

Supplementary Dataset 5

Supplementary Dataset 6

Supplementary Dataset 7

Supplementary Dataset 8

Supplementary Dataset 9

Supplementary Dataset 10

Supplementary Dataset 11

Supplementary Dataset 12

Supplementary Dataset 13

Supplementary Dataset 14

Reporting Summary

## Data Availability

All crystal structures used in this study were retrieved from the Protein Data Bank (PDB) and can be accessed at https://www.rcsb.org. The accession codes of the structures in the ideal dataset are: 1IZ4, 1JMA, 1K7G, 1KB0, 1KTU, 1LI1, 1N7V, 1NLT, 1OKC, 1QHU, 1QQC, 1R3F, 1RNE, 1SUU, 1T9F, 1YNF, 1YU0, 1ZVC, 2F3L, 2FM9, 2GD5, 2ISB, 2JAI, 2P8H, 2QGQ, 2QSD, 2ZZQ, 3A1Y, 3BXO, 3C7X, 3CPW, 3E8T, 3EB7, 3EF6, 3ENI, 3G91, 3GUA, 3KYJ, 3M7M, 3N99, 3ODJ, 3P0Y, 3RST, 3S2O, 3SAE, 3VZ9, 3WPV, 4AI1, 4D6F, 4E7G, 4I1M, 4QMJ, 4XTK, 5KIS, 5LB7, 5MIN, 5U69, 5VSK, 6AZZ, 6S2M. The accession codes of the structures in the experimental dataset are: 1BEB, 1BN1, 1CFD, 1DPX, 1EX3, 1FD3, 1FS3, 1HFX, 1HRC, 1J7N, 1LDS, 1LFG, 1OVA, 1QOY, 1UBQ, 1VXG, 1YTQ, 2MLT, 3INS, 3QYT, 3VWI, 4F5S, 4H2A, 6DAH, 6PTI. The CATH Protein Structure Classification database was used to determine the architectures of proteins and can accessed here: http://www.cathdb.info. The processed simulation data in this work are available without any restriction from GitHub (https://github.com/smturzo/IMMS/tree/v.1.0.0) and Zenodo^75^ (10.5281/zenodo.6726418). Additionally, our GitHub repository contains instructions on how this work can be reproduced. Access to raw simulation data (not present in this repository due to size limitation) can be obtained by emailing the corresponding author (lindert.1@osu.edu). Processed simulation data can also be found in the Supplementary Data files. Source data are provided with this paper.

## Source data

Source Data

## Code availability

The Collision Cross Section application (PARCS v.1.0.0) and the Ion Mobility score function are available for free to academic users through the Rosetta software suite at https://www.rosettacommons.org/software/. The current academic version of Rosetta (3.13) can be freely downloaded from https://els2.comotion.uw.edu/product/rosetta for academic users. The source code for PARCS and the Ion Mobility score function (which are part of the Rosetta codebase) are only made available to academic/non-profit/government entities and commercial entities with a Company Contributor License. While availability to the Rosetta codebase is free for academics/non-profit/government entities, note that there is a Rosetta license fee for industry users to gain access to the source code and the applications in Rosetta (including the PARCS application and the Ion Mobility score function). Currently the University of Washington exclusively manages all Rosetta licensing. More information on Rosetta licensing can be found at https://www.rosettacommons.org/about/faq. Instructions to run the PARCS application in Rosetta can be found in Supplementary Note 1. Instructions on protein structure prediction with IM data can be found in Supplementary Note 2. Both of these instructions are accompanied by a step-by-step example. Additionally, instructions and results of modifying the AlphaFold source code such that it predicts models without the aid of templates can be found in Supplementary Note 3 and Supplementary Data 14, respectively.
