# Supplementary material for: Protein shape sampled by ion mobility mass spectrometry consistently improves protein structure prediction
Source: Nat Commun. 2022 Jul 28;13:4377. doi: 10.1038/s41467-022-32075-9 (PMC9334640; doi:10.1038/s41467-022-32075-9)
Supplement: Supplementary file 1 — Supplementary Information [file 41467_2022_32075_MOESM1_ESM.pdf]

Supplementary Information: Protein shape sampled by ion mobility mass spectrometry consistently improves protein structure prediction

SM Bargeen Alam Turzo[1], Justin T. Seffernick[1], Amber D. Rolland[2], Micah T. Donor[2], Sten Heinze[1], James S. Prell[2], Vicki Wysocki[1] and Steffen Lindert[1, *]

[1]Department of Chemistry and Biochemistry and Resource for Native Mass Spectrometry Guided Structural Biology, Ohio State University, Columbus, OH, 43210
[2]Department of Chemistry and Biochemistry and Materials Science Institute, University of Oregon, Eugene, OR, 97403

* Correspondence to:
Department of Chemistry and Biochemistry, Ohio State University
2114 Newman & Wolfrom Laboratory, 100 W. 18th Avenue, Columbus, OH 43210
614-292-8284 (office), 614-292-1685 (fax)
lindert.1@osu.edu

Supplementary Methods

Procedures for *ab initio* and comparative modelling protocol in Rosetta, AlphaFold2, RoseTTAFold, RG score function, model quality assessment programs and simulated noise in the ideal dataset

The PDB structures of Lethal Factor N-Terminus (1J7N), Cytolysin A (1QOY), β-crystallin B2 (1YTQ), Fragaceatoxin C (3VWI) and Protective Antigen 63 (4H2A) from the experimental dataset had missing and/or extra residues compared to the protein under experimental IM conditions. For these structures, to ensure that our native structure corresponded to the exact same sequence that was used in the IM measurements, coordinates for missing residues at the C and N termini (CT and NT respectively) were built, as outlined in Supplementary Data 13, using a modified CM protocol in Rosetta[1-7].

For the ideal and experimental data sets as shown in Supplementary Data 1 and Supplementary Data 6 respectively, a decoy set of 10,000 structures was generated for each protein using the AbinitioRelax and comparative modeling (CM) algorithms (as appropriate) within Rosetta. In general, CM outperforms *ab initio* modeling. This is because CM is template-based modeling, whereas *ab initio* is template free modeling. Therefore, if templates with high sequence identity and coverage to the target are found then CM likely predicts structures that are generally close to the native structure. On the other hand, *ab initio* structure prediction does not use any template information. Therefore, the *ab initio* protocol generates a diverse set of predictions. Thus, selecting the model that most closely represents the native structure depends more extensively on the physics and knowledge-based score function. Extensive details about ab initio and CM protocol can be found elsewhere[8].

All structures were generated by parallelizing jobs over 100 CPU cores on the Ohio Supercomputer Center[9]. The time required for generating and scoring 10,000 structures scaled with the number of residues. For example, times required to generate 10,000 models for sequence lengths of 58, 103, 304 were approximately 3, 7 and 28 hours, respectively when parallelized over 100 CPU cores.

All fragments (required for both CM and *ab initio*) were generated using the fragment picker tool [10]. For systems where the ab initio protocol was used (Supplementary Data 1 and Supplementary Data 6) for generating the decoy sets, the required fragments were generated by excluding homologs. However, for cases of poor sampling, where the minimum RMSD of the decoy set was greater than to 7 Å, the fragments were re-generated by including homologs. And the decoy set of 10,000 structures was built again with the new fragments.

For systems that required the CM protocol (Supplementary Data 1 and Supplementary Data 6), templates (and their weights) were chosen (Supplementary Data 11 and Supplementary Data 12) such that a broad RMSD (and TM-Score) distribution was obtained with respect to the native protein. This was done because presence of both native-like and non-native-like models was necessary for benchmarking purposes to demonstrate the ability of CCS data to distinguish between good and bad models.

A proxy score function was also developed with radius of gyration (RG) to favor compact models. This score function selected models with lowest RG as the best scoring model from a set of structures. All generated structures (from *ab initio* and CM) were subjected to the Rosetta Relax protocol using the Rosetta energy function (RS)[11], RG and the IM score functions. The lowest scoring models were then designated as the final models in all above cases.

All structures in both the ideal and experimental dataset were also predicted with AlphaFold2[12] (AF) and RoseTTAFold[13] (RF) with default settings with and without templates. By default, both methods predict structures with the aid of templates. For AF, to predict with templates the maximum template date was set to one day before the deposited date of the PDB (as per instructions in https://github.com/deepmind/alphafold). This ensured that the benchmark structure itself was not used as template. However, other structures (deposited before the target) were used as templates. The same was ensured for RF by removing the benchmark PDBs from the template database. There is no direct way to predict structures without templates in AF, therefore we employed two different methods to achieve the same goal (as outlined here). The first method was to set the maximum template date to 1900-01-01. In this method, AF searched for templates from the database, however setting the maximum template date this far back effectively ensured that no templates were found in the PDB database (the PDB database was created in 1971). The second method to ignore templates in AF was to make slight changes in the AF source code as outlined in Supplementary Note 3 of this document. In the data shown, we used the first method to remove all templates for AF predictions, however in Supplementary Data 14 we show that the RMSD and TM-Score from both these options were practically identical. For RF, protein structures without templates were predicted by omitting the option "—atab" when running the prediction.

For the experimental dataset, the native structures (ground truth PDBs) used to compare to the predicted models were chosen such that the sequence and experimental conditions most closely resembled the IM experiment (Supplementary Data 2).

Model quality of all best scoring models from the IM score function was further assessed with Voronota[14,15] and P3CMQA[16] (in their default settings) for both the ideal and experimental dataset. To better compare the IM Confidence score to Voronota and P3CMQA, the IM Confidence score was scaled by dividing the confidence score by the most negative confidence score from each dataset (ideal and experimental).

Random amounts of noise were generated in CCS_{Ideal}. To create noise for the ideal noise, $\chi\%$ noise was randomly selected anywhere from - $\gamma\%$ to + $\gamma\%$ and added to CCS_{Ideal} of proteins in the ideal dataset as shown in Supplementary Equation 1. These CCS_{Ideal} with noise were then used to score structures in the ideal dataset.

$$\begin{gathered} CCS_{Ideal\_with\_\gamma\%\_noise} = CCS_{Ideal} + (CCS_{Ideal} \times \chi\%) \\ where\ \gamma = [15, 30] \\ and\ -\gamma \leq \chi \leq +\gamma \end{gathered} \quad (1)$$

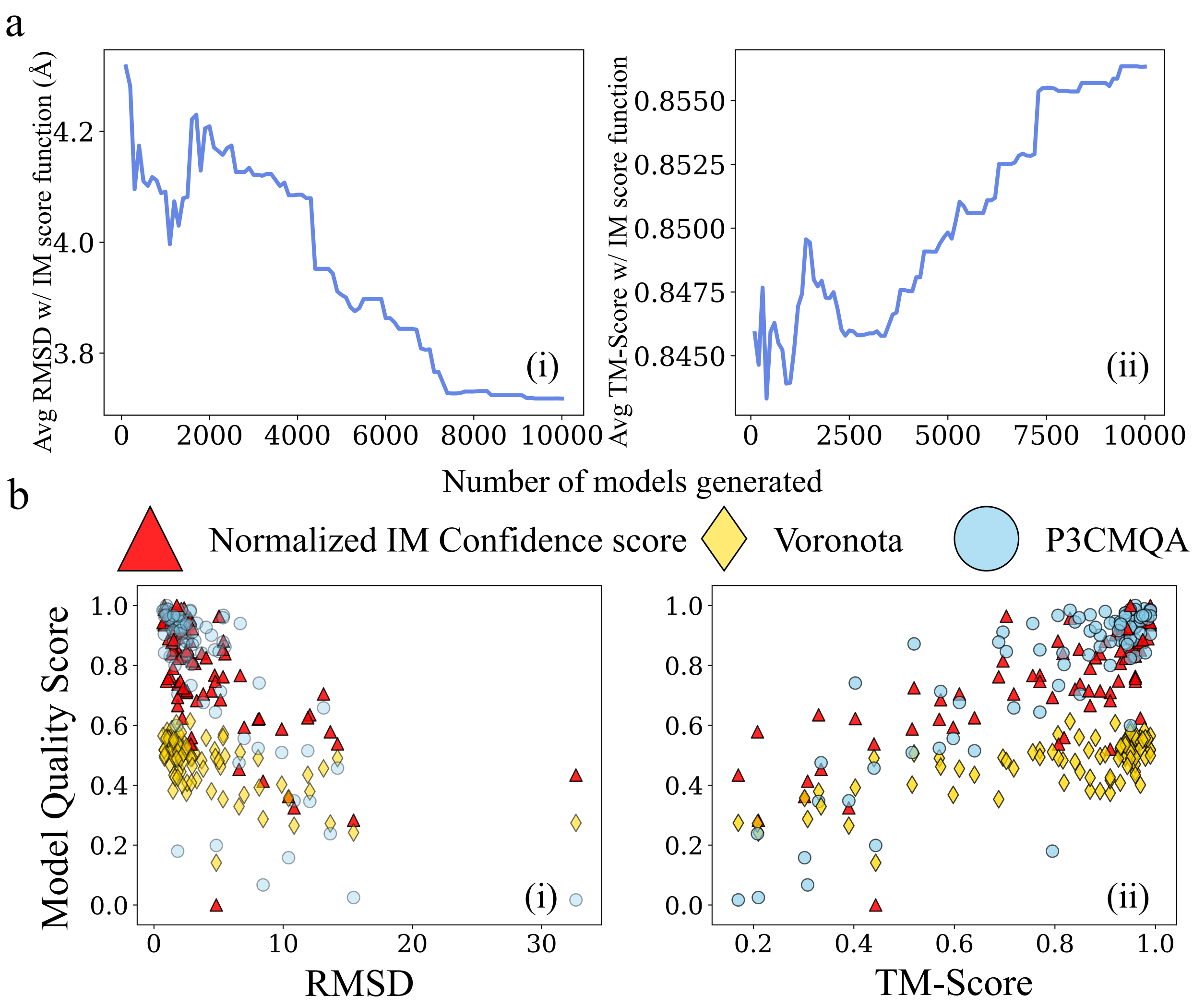

Supplementary Figure 1. Sampling size and model quality with ion mobility (IM) data. Prediction with the IM score function improves with increased sampling. (a) Comparison of the (i) average root mean square deviation (RMSD) and (ii) average template modelling score (TM-Score) of the best scoring model improved effectively when the number of generated decoys was increased from 100 to 10,000 for the ideal dataset. (b) Comparison of model quality score for normalized IM confidence score (red triangle), Voronota (yellow diamond), and P3CMQA (blue circle) for proteins in both (experimental and ideal) datasets against their respective (i) RMSD and (ii) TM-Score. Source data are provided as a Source Data file.

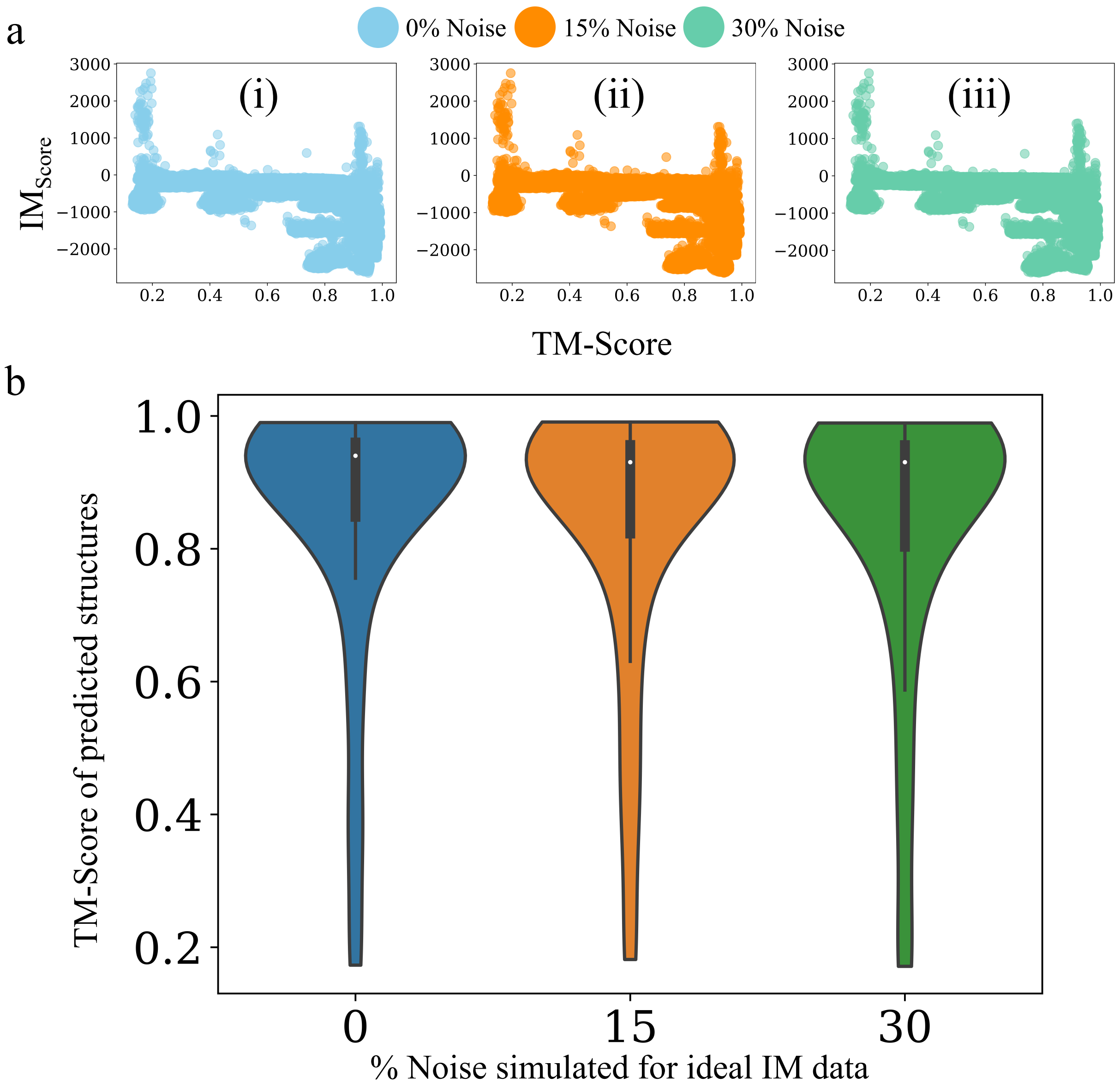

Supplementary Figure 2. Prediction results with noisy collision cross sections simulated from the ideal dataset (CCS_{Ideal}) data. Random noise at 15% (orange) and 30% (green) was added to CCS_{Ideal} (no noise, blue) for all generated structures (600000 structures) in the ideal dataset. In comparison to (i) 0% noise, the IM score vs TM-Score distribution with (ii)15%, and 30% (iii) noise showed no significant change. (b) In these violin distributions (n = 600000 biologically independent samples over 3 independent random noise simulations), no significant change in the global folds was observed for the best scoring models when random noise was introduced at 15% and 30% as compared to those with 0% noise. The mean and the standard error of mean of TM-Score for the distributions in (b) are 0.857 ± 0.024, 0.841 ± 0.025, and 0.839 ± 0.026 respectively. The white dots represent the median in each violin distribution. The black bar in the center of the distribution is the interquartile range (IQR). The black stretched line extends from the “first quartile – 1.5 IQR”

to the “third quartile + 1.5 IQR”. Values beyond this range are considered outliers. Source data are provided as a Source Data file.

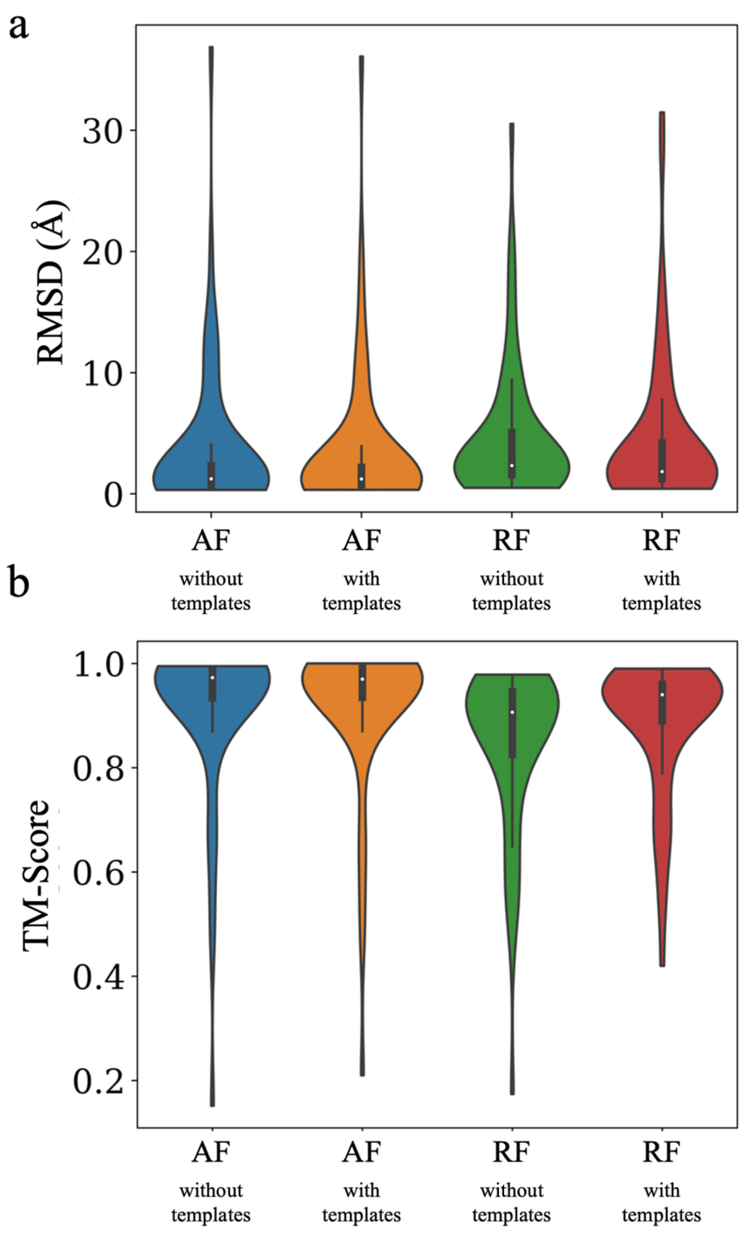

Supplementary Figure 3. Comparison of prediction results for the ideal dataset with AlphaFold (AF) and RosettaFold (RF). The prediction results from AF and RF (both with and without templates) are compared by (a) root mean square deviation (RMSD) and (b) template modelling score (TM-Score) with n = 60 biologically independent samples over 4 independent modelling approaches. For the predictions methods in (a) the mean and the standard error of mean are 3.64 ± 0.79 Å, 3.47 ± 0.77 Å, 4.70 ± 0.73 Å, and 4.37 ± 0.80 Å respectively. Similarly, these values in (b) are 0.91 ± 0.02, 0.92 ± 0.02, 0.85 ± 0.02, and 0.88 ± 0.01 respectively. For the violin distributions in (a) and (b) the white dots represent the median. The black bar in the center of the distribution is the interquartile range (IQR). The black stretched line extends from the "first quartile – 1.5 IQR" to the "third quartile + 1.5 IQR". Values beyond this range are considered outliers. Source data are provided as a Source Data file.

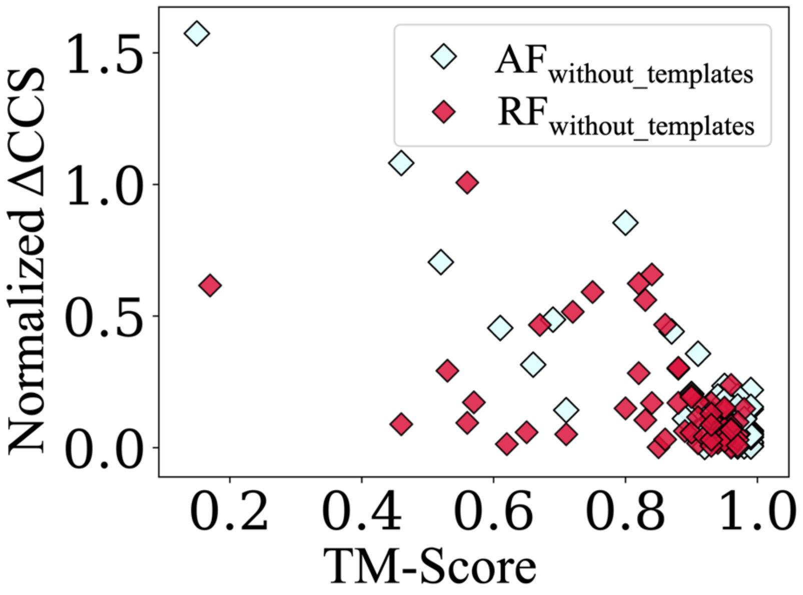

Supplementary Figure 4. Poorly folded structures by AlphaFold (AF) and RosettaFold (RF) are penalized by comparison to ion mobility (IM) data. High normalized absolute difference in collision cross section of the predicted structure and the native structure (ΔCCS divided by sequence length) for structures predicted with AF without templates ($AF_{without\_templates}$,cyan) and RF without templates ($RF_{without\_templates}$,red) generally corresponded to structures with low template modelling score (TM-Score) as seen for the ideal dataset. Source data are provided as a Source Data file.

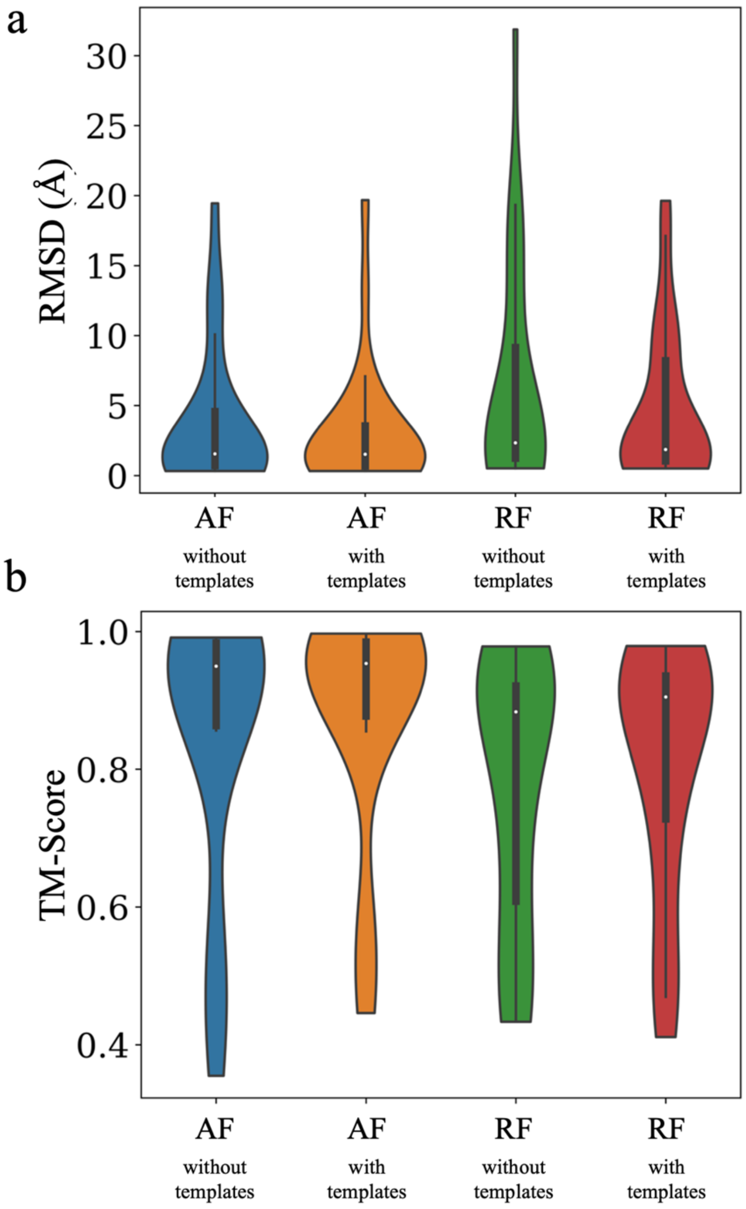

Supplementary Figure 5. Comparison of prediction results for the experimental dataset with AlphaFold (AF) and RosettaFold (RF). The prediction results from AF and RF (both with and without templates) are compared by (a) root mean square deviation (RMSD) and (b) template modelling score (TM-Score) with n = 25 biologically independent samples over 4 independent modelling approaches. The mean and the standard error of mean for the methods in (a) are 3.70 ± 1.01 Å, 3.13 ± 0.90 Å, 6.51 ± 1.63 Å, and 4.57 ± 1.08 Å respectively. Similarly these values for (b) are 0.85 ± 0.04, 0.88 ± 0.03, 0.78 ± 0.04, and 0.81 ± 0.03 respectively. For the violin distributions in (a) and (b) the white dots represent the median. The black bar in the center of the distribution is the interquartile range (IQR). The black stretched line extends from the "first quartile – 1.5 IQR" to the "third quartile + 1.5 IQR". Values beyond this range are considered outliers. Source data are provided as a Source Data file.

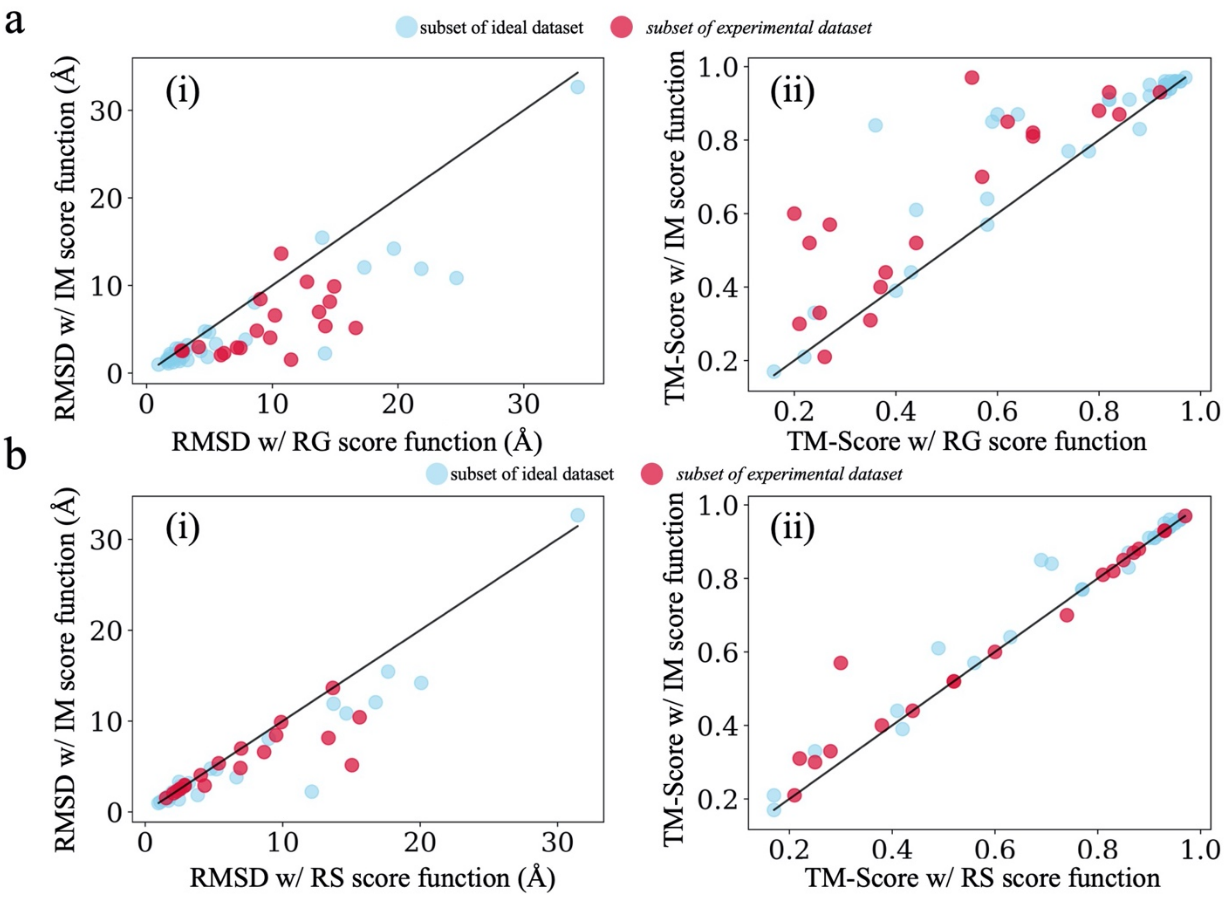

Supplementary Figure 6. Improved structure prediction with ion mobility (IM) data for sequences with poor or no templates. Consistent improvement in model selection was observed when using the IM score function for the subset of 54 proteins where comparative modelling (CM, with non-perfect templates) and *ab initio* (template-free) protocols were utilized. The predicted models from the IM score function were compared to those of the (a) radius of gyration (RG) and (b) Rosetta (RS) score functions in terms of their respective (i) root mean square deviation (RMSD) and (ii) template modelling score (TM-Score). For both (a) and (b) the subset of models from the ideal and experimental dataset are shown in blue and red respectively. Source data are provided as a Source Data file.

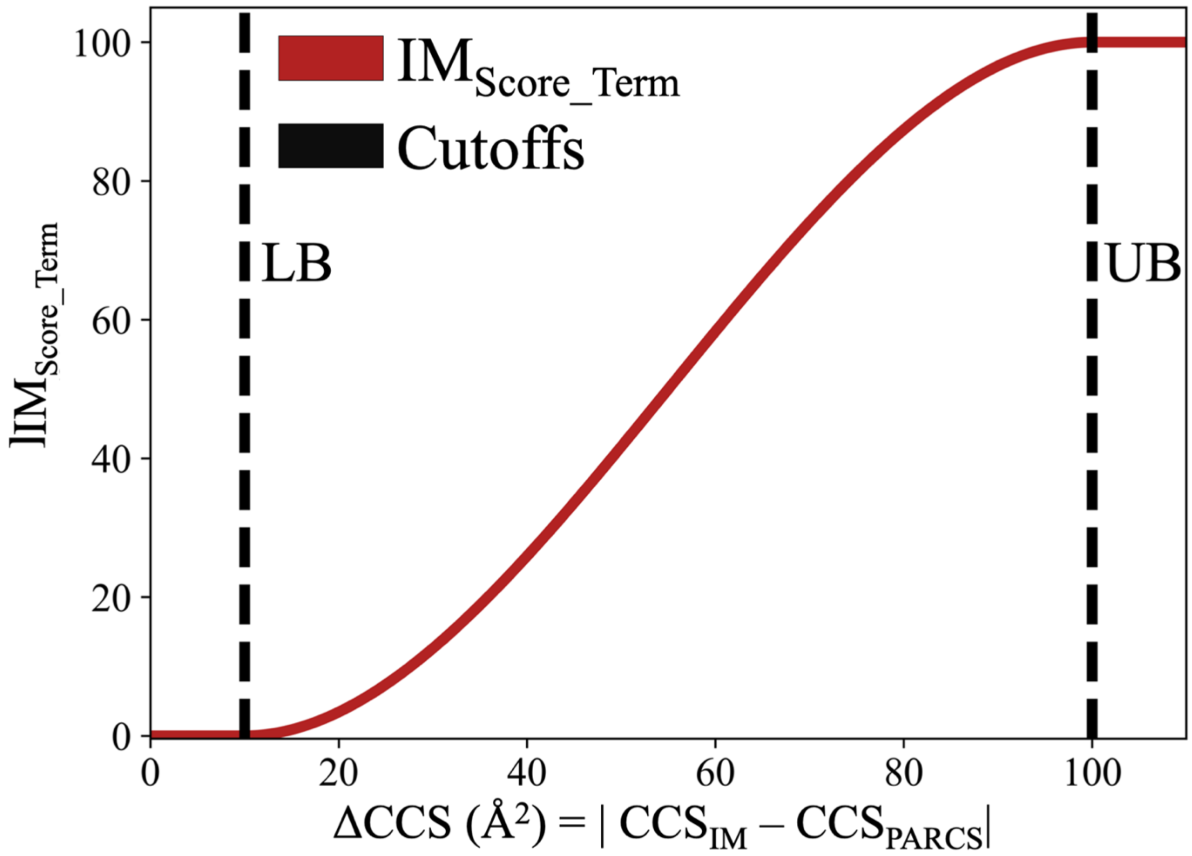

Supplementary Figure 7. Illustration of the ion mobility score term (IM_{Score_Term}). IM_{Score_Term} is a fade function where LB and UB are the lower and upper bound cutoffs set at 10 Å^2 and 100 Å^2, respectively. This term penalizes structures based on the absolute difference between the experimental collision cross section (CCS) and the structures' predicted CCS. Source data are provided as a Source Data file.

Supplementary Note 1:

General usage of PARCS application

A structure is required to run this application. To use PARCS to predict the CCS of given structure(s), users need to specify the full path to the executable of the PARCS application (<path/to/Rosetta>/main/source/bin/parcs_ccs_application.default.<os><compiler>release). User also need to provide the full path to Rosetta database with the flag *-database*. Next, the structure of the protein for which CCS is to be predicted is specified with *-in:file:s* (or *-in:file:l* for list of structures) in a format readable by Rosetta. Users may choose to specify the number of rotations with *-ccs_nrots* (default is set to 300). Probe radius is set to 1.0 Å by default to predict CCS in helium buffer gas. The other option is to set it to 1.81 Å by using option *-ccs_prad* to predict CCS in nitrogen buffer gas. By default, the application will save the output containing two pieces of information (the name of the structure file and CCS value in Å^2) to a file named 'CCS_default.out'. However, users can define the output file name with the flag *-out:file:o*. General usage of the command-line option to run CCS calculation on a single structure is shown below, where variables that need to be specified by users are shown in brackets (< >) and are defined below:

<path/to/Rosetta>/main/source/bin/parcs_ccs_calc.default.<os><compiler>release *-database* <path/to/Rosetta>/main/database *-in:file:s* <structure> *-ccs_nrots* <number_of_rotations> *-ccs_prad* <probe_radius_in_angstroms> *-out:file:o* <output_file_name>

- path/to/Rosetta – Users' path to Rosetta
- os – Name of operating system (linux, mac, etc.)
- compiler – Name of C++ compiler (gcc, clang, etc.)
- structure – Name of structure for which CCS calculation is performed.
- number_of_rotations – Number of random rotations for CCS calculations. Must be an integer. Default is set to 300.
- probe_radius_in_angstroms – Radius of the buffer gas probe. Default is set to 1.0 Å for helium gas. For nitrogen gas please use 1.81 Å.
- output_file_name – User-defined output file name. Default is set to "CCS_default.out".

Example usage of PARCS application to predict CCS of ubiquitin (1UBQ)

In this example the CCS of ubiquitin, with a crystal structure available in the PDB (1UBQ), is predicted with PARCS. Note: In this tutorial we will assume that our operating system is Linux and our compiler is gcc.

1. Create a new directory for input and output files and enter this directory

> mkdir calculate_ccs_for_known_structure && cd calculate_ccs_for_known_structure

2. Download the crystal structure of ubiquitin from https://www.rcsb.org/structure/1UBQ into the calculate_ccs_for_known_structure directory

3. PDB files often contain other useful information, such as water molecules, non-standard amino acids, additional molecules pertaining to experimental conditions, etc. However, this extra information may cause Rosetta to fail if the input structure file is not properly prepared. Fortunately, Rosetta offers a python script (clean_pdb.py) to work around this

issue. Use this python script on the PDB file and specify the file name and chain of interest. For ubiquitin this is chain A and the command is.

> python <path/to/Rosetta>/tools/protein_tools/scripts/clean_pdb.py 1UBQ.pdb A

Note: The script clean_pdb.py should produce two files. These are 1UBQ_A.fasta and 1UBQ_A.pdb. For this tutorial only 1UBQ_A.pdb file is utilized.

4. Predict the CCS with 250 random rotations and a probe radius of 1.0 and save output file as ‘1ubq_predicted_ccs.txt’ with the following command.

> <path/to/Rosetta>/main/source/bin/parcs_ccs_calc.default.linuxgccrelease -*database* <path/to/Rosetta>/main/database -*in:file:s* 1UBQ_A.pdb -*ccs_nrots* 250 -*ccs_prad* 1.0 -*out:file:o* 1ubq_predicted_ccs.txt’

5. 1ubq_predicted_ccs.txt contains two pieces of information, the name of the file and CCS value in Å^2 as shown below.

File_Name	CCS_PARCS
1UBQ.pdb	927.547

<u>Supplementary Note 2</u>:

General usage of scoring (using IM score function) predicted structures with IM data

This tutorial explains how to score structures (obtained from *ab initio* or CM protocol in Rosetta) with IM data (using IM score function). The users need to provide the full path to the score application (<path/to/Rosetta>/main/source/bin/score.default.<os><compiler>release). Full path to database with the flag *-database* is also required. The users also need to specify the structure generated from the prediction protocol with the flag *-in:file:s* (or *-in:file:l* for list of generated structures). The probe radius (required to predict CCS of structures for use in the score function) is set to 1.0 Å by default for helium buffer gas conditions and can be changed to 1.81 Å (with the flag *-ccs_prad*) if the IM experiment was carried out in nitrogen buffer gas conditions. The number of random rotations (*-ccs_nrots*) is set to 300 by default and can be changed as needed. The experimental CCS, derived from IM for the protein of interest, is provided with the required flag *-ccs_exp*. Users also need to specify the patch file (with the option *-score:patch*) *ccs_imms.wts_patch* that calls the IM score function. A general usage of this score function is shown below, where the variables that need to be specified by users are shown in brackets (< >) and are defined below:

<path/to/Rosetta>/main/source/bin/score.default.<os><compiler>release *-database* <path/to/Rosetta>/main/database *-in:file:s* <structure_from_prediction_protocol> *-ccs_nrots* <number_of_rotations> *-ccs_prad* <probe_radius_in_angstroms> *-ccs_exp* <experimental_ccs_data> *-score:patch* ccs_imms.wts_patch

- path/to/Rosetta – Users' path to Rosetta
- os – Name of operating system (linux, mac, etc.)
- compiler – Name of C++ compiler (gcc, clang, etc.)
- structure_from_prediction_protocol – Structures generated either by using the *ab initio* or CM protocol in Rosetta.
- number_of_rotations – Number of random rotations for CCS calculations. Must be an integer. Default is set to 300.
- probe_radius_in_angstroms – Radius of the buffer gas probe. Default is set to 1.0 Å for helium gas. For nitrogen gas please use 1.81 Å.
- experimental_ccs_data – Experimental CCS value determined from IM experiments (in Å^2). If CCS_{IM} is determined from nitrogen buffer gas, set *-ccs_prad* to 1.81 Å. If CCS data is from helium gas, then by default *-ccs_prad* is set to 1.00 Å.

Example usage of scoring decoy structures (from *ab initio* protocol) of ubiquitin (1UBQ) with IM data

This tutorial uses ubiquitin (PDB ID: 1UBQ) as an example. A known structure is not required for model generation but providing a native structure will result in RMSD calculation. In this tutorial we will also assume that our operating system is Linux and our compiler is gcc.

1. Create a new directory for input and output files and enter this directory.
   > mkdir score_with_im_data && cd score_with_im_data
2. Download crystal structure of ubiquitin from https://www.rcsb.org/structure/1UBQ in PDB file format.
3. Prepare the file for use with Rosetta.
   > python <path/to/Rosetta>/tools/protein_tools/scripts/clean_pdb.py 1UBQ.pdb A

4. Use Robetta websever (http://old.robetta.org/fragmentsubmit.jsp) to generate the 3mer, 9mer and secondary structure prediction (recommended). Alternatively use fragment picker tool (if set up correctly) to generate these files with this command.

> <path/to/Rosetta>/tools/fragment_tools/make_fragments.pl -verbose -nohoms 1UBQ_A.fasta

Note: The fragment picker tool will generate many files, but the files that are utilized from this step are t001_.200.3mers, t001_.200.9mers and t001_.psipred_ss2.

5. Create a flags file (1ubq_abinitio_flags) for the *ab initio* structure generation protocol with these flags.

-in:file:fasta 1UBQ_A.fasta
-in:file:frag3 t001_.200.3mers
-in:file:frag9 t001_.200.9mers
-psipred_ss2 t001_.psipred_ss2
-nstruct 10
-abinitio:relax
-use_filters true
-abinitio::increase_cycles 10
-abinitio::rg_reweight 0.5
-abinitio::rsd_wt_helix 0.5
-abinitio::rsd_wt_loop 0.5
-ex1
-ex2aro
-relax::fast
-out:file:silent ./fold_silent.out

6. Run the AbinitioRelax command on the terminal window with this command.

> <path/to/Rosetta>/main/source/bin/AbinitioRelax.linuxgccrelease *-database* <path/to/Rosetta>/main/database @1ubq_abinitio_flags

7. The flag "*-out:file:silent*" in the 1ubq_abinitio_flags file instructs Rosetta to store all 10 structures in a file named fold_silent.out file. When structure generation is complete, run the Rosetta Relax protocol to relax all 10 structures and save them as individual pdbs with this command.

> <path/to/Rosetta>/main/source/bin/relax.linuxgccrelease *-database* <path/to/Rosetta>/main/database *-in:file:silent* fold_silent.out *-in:file:fullatom -relax:quick -nstruct* 1 *-out:prefix* r_

Note: More structures can be generated by increasing the number associated with the flag "*-nstruct*" in the 1ubq_abinitio_flags file.

8. Store the names of all 10 'relaxed' structures (with all output files having the prefix 'r_' because of the flag "*-out:prefix*") generated from Relax protocol with this command.

> ls r_*.pdb > structurelist.txt

9. Run the score application with this command.

> <path/to/Rosetta>/main/source/bin/score.default.linuxgccrelease *-database* <path/to/Rosetta>/main/database *-in:file:l* structurelist.txt *-ccs_nrots* 250 *-ccs_prad* 1.0 *-ccs_exp* 930 *-score:patch* ccs_imms.wts_patch *-in:file:native* 1UBQ_A.pdb

Note: The flag *-in:file:native* is optional and is used for RMSD calculation.

10. The 'default.sc' file produced by the score application contains a lot of information including the IM term (ccs_imms) that contributed to

the IM score (score), RMSD (rms) and the decoy structure (description) that corresponded to this information as shown below.

SCORE:	score	...	ccs_imms	...	rms	description
SCORE:	-182.443	...	7.046	...	5.691	r_F_00000005_0001_0001
SCORE:	-145.027	...	31.242	...	11.382	r_F_00000006_0001_0001
SCORE:	-187.302	...	12.786	...	3.204	r_S_00000001_0001_0001
SCORE:	-188.296	...	11.096	...	4.594	r_S_00000002_0001_0001
SCORE:	-105.458	...	71.117	...	9.342	r_S_00000003_0001_0001
SCORE:	-183.859	...	0.018	...	4.850	r_S_00000004_0001_0001
SCORE:	-206.509	...	0.000	...	4.049	r_S_00000007_0001_0001
SCORE:	-40.841	...	100.000	...	11.913	r_S_00000008_0001_0001
SCORE:	-44.949	...	100.000	...	9.460	r_S_00000009_0001_0001
SCORE:	-137.448	...	15.016	...	3.404	r_S_00000010_0001_0001

Note: Other terms not important to this tutorial are represented as '…'

11. The IM score and RMSD of each 'relaxed' structure compared to the native of the generated structure is extracted from the 'default.sc' file (produced by score application) with the following command.

```
> python
    > import numpy as np, pandas as pd, matplotlib.pyplot as plt
    > score_file  = pd.read_csv('default.sc', sep='\s+', header=0)
    > score        = score_file['score']
    > rmsd         = score_file['rms']
    > plt.figure()
    > plt.scatter(rmsd,score,color='pink')
    > plt.xlabel(r'RMSD ($\AA$)')
    > plt.ylabel('Score with experimental IM data')
    > plt.savefig('1UBQ_SCORE_VS_RMSD.png',dpi=300)
    > plt.close()
```

12. View results in file '1UBQ_SCORE_VS_RMSD.png'.

Note: This plot is only meaningful when a large number of structures are generated.

<u>Supplementary Note 3</u>

Predicting protein structures without templates by modifying the source code in AF

To predict protein structure in AF without the aid of templates (using the option 2, as explained before), users need to modify the config.py file within AF source code. The path to this file is at https://github.com/deepmind/alphafold/blob/main/alphafold/model/config.py.

In this file, the users need to change the following boolean **True** to **False** (shown in highlight):

```
CONFIG_DIFFS = {

    'model_1': {
        # Jumper et al. (2021) Suppl. Table 5, Model 1.1.1
        'data.common.max_extra_msa': 5120,
        'data.common.reduce_msa_clusters_by_max_templates': False,
        'data.common.use_templates': False,
        'model.embeddings_and_evoformer.template.embed_torsion_angles': False,
        'model.embeddings_and_evoformer.template.enabled': False
    },
    'model_2': {
        # Jumper et al. (2021) Suppl. Table 5, Model 1.1.2
        'data.common.reduce_msa_clusters_by_max_templates': False,
        'data.common.use_templates': False,
        'model.embeddings_and_evoformer.template.embed_torsion_angles': False,
        'model.embeddings_and_evoformer.template.enabled': False
    }
.
.
.
    'model_1_ptm': {
        'data.common.max_extra_msa': 5120,
        'data.common.reduce_msa_clusters_by_max_templates': False,
        'data.common.use_templates': False,
        'model.embeddings_and_evoformer.template.embed_torsion_angles': False,
        'model.embeddings_and_evoformer.template.enabled': False,
        'model.heads.predicted_aligned_error.weight': 0.1
    },
    'model_2_ptm': {
        'data.common.reduce_msa_clusters_by_max_templates': False,
        'data.common.use_templates': False,
        'model.embeddings_and_evoformer.template.embed_torsion_angles': False,
        'model.embeddings_and_evoformer.template.enabled': False,
        'model.heads.predicted_aligned_error.weight': 0.1
    }
```

Supplementary References

1. Simons, K. T., Kooperberg, C., Huang, E. & Baker, D. Assembly of protein tertiary structures from fragments with similar local sequences using simulated annealing and Bayesian scoring functions. *J Mol Biol* **268**, 209-225 (1997). https://doi.org:10.1006/jmbi.1997.0959
2. Simons, K. T. *et al.* Improved recognition of native-like protein structures using a combination of sequence-dependent and sequence-independent features of proteins. *Proteins: Structure, Function, and Bioinformatics* **34**, 82-95 (1999). https://doi.org:10.1002/(sici)1097-0134(19990101)34:1<82::aid-prot7>3.0.co;2-a
3. Bonneau, R. *et al.* Rosetta in CASP4: progress in ab initio protein structure prediction. *Proteins* **Suppl 5**, 119-126 (2001). https://doi.org:10.1002/prot.1170
4. Bonneau, R. *et al.* De novo prediction of three-dimensional structures for major protein families. *J Mol Biol* **322**, 65-78 (2002). https://doi.org:10.1016/s0022-2836(02)00698-8
5. Bradley, P., Misura, K. M. & Baker, D. Toward high-resolution de novo structure prediction for small proteins. *Science* **309**, 1868-1871 (2005). https://doi.org:10.1126/science.1113801
6. Leaver-Fay, A. *et al.* ROSETTA3: an object-oriented software suite for the simulation and design of macromolecules. *Methods in enzymology* **487**, 545-574 (2011). https://doi.org:10.1016/B978-0-12-381270-4.00019-6
7. Raman, S. *et al.* Structure prediction for CASP8 with all-atom refinement using Rosetta. *Proteins* **77 Suppl 9**, 89-99 (2009). https://doi.org:10.1002/prot.22540
8. Bender, B. J. *et al.* Protocols for Molecular Modeling with Rosetta3 and RosettaScripts. *Biochemistry* **55**, 4748-4763 (2016). https://doi.org:10.1021/acs.biochem.6b00444
9. (1987).
10. Gront, D., Kulp, D. W., Vernon, R. M., Strauss, C. E. & Baker, D. Generalized fragment picking in Rosetta: design, protocols and applications. *PLoS One* **6**, e23294 (2011). https://doi.org:10.1371/journal.pone.0023294
11. Alford, R. F. *et al.* The Rosetta All-Atom Energy Function for Macromolecular Modeling and Design. *Journal of Chemical Theory and Computation* **13**, 3031-3048 (2017). https://doi.org:10.1021/acs.jctc.7b00125
12. Jumper, J. *et al.* Highly accurate protein structure prediction with AlphaFold. *Nature* **596**, 583-589 (2021). https://doi.org:10.1038/s41586-021-03819-2
13. Baek, M. *et al.* Accurate prediction of protein structures and interactions using a three-track neural network. *Science* **373**, 871-876 (2021). https://doi.org:doi:10.1126/science.abj8754
14. Olechnovič, K. & Venclovas, C. Voronota: A fast and reliable tool for computing the vertices of the Voronoi diagram of atomic balls. *J Comput Chem* **35**, 672-681 (2014). https://doi.org:10.1002/jcc.23538
15. Olechnovič, K. & Venclovas, Č. VoroMQA: Assessment of protein structure quality using interatomic contact areas. *Proteins* **85**, 1131-1145 (2017). https://doi.org:10.1002/prot.25278
16. Takei, Y. & Ishida, T. P3CMQA: Single-Model Quality Assessment Using 3DCNN with Profile-Based Features. *Bioengineering* **8**, 40 (2021).
