# Supplementary material for: Protein shape sampled by ion mobility mass spectrometry consistently improves protein structure prediction
Source: Nat Commun. 2022 Jul 28;13:4377. doi: 10.1038/s41467-022-32075-9 (PMC9334640; doi:10.1038/s41467-022-32075-9)
Supplement: Supplementary file 2 — Description of Additional Supplementary Files [file 41467_2022_32075_MOESM2_ESM.docx]

**Description of Additional Supplementary Files**

File Name: Supplementary Data 1

Description: Description of the ideal dataset. Protein structures from the ideal dataset (PDB ID) with their corresponding chain ID, sequence length as well as their class, architecture, and domain ID as defined by the protein structure classification database CATH, and modelling protocol (Method) that was utilized to generate decoys per structure.

File Name: Supplementary Data 2

Description: Benchmarking results of the ideal dataset. Analysis of best scoring models of protein structures from ideal dataset (PDB ID) to their native structures. These structures were predicted with radius of gyration score function (RG RMSD & RG TM-Score), Rosetta score function (RS RMSD & RS TM-Score), Ion Mobility score function (IM RMSD & IM TM-Score), AlphaFold2 (AF_with_templates_ RMSD & AF_with_templates_ TM-Score) and RoseTTAFold (RF_with_templates_ RMSD & RF_with_templates_ TM-Score). Comparison showing the improvement of energy funnel when IM score function was utilized in place of RG (IM_P_near_: RG_P_near_) and RS (IM_P_near_: RS_P_near_) score function.

File Name: Supplementary Data 3

Description: Compaction analysis of ideal dataset with simulated ion mobility data. Comparison of compactness (as measured by R_g_) and side chain collapse (as measured by neighbor count, NC) of models predicted with the IM score function when CCS_Ideal_ was reduced (% CCS_Ideal_ reduced by). The average Rg (Avg. R_g_ [Å]), average RMSD (Avg. RMSD [Å]), average TM-Score (Avg. TM-Score), and the average NC of top X % residues with low neighbor count (Avg. NC of top X % residues) are shown, where X ranges from 5 to 20 for all 60 predicted models at varying reduced CCS_Ideal_. The Avg. R_g_ and Avg. NC of top X% residues of the native crystal structures (Native) are given as reference. Source data are provided as a Source Data file.

File Name: Supplementary Data 4

Description: Comparison of CCS predictions with different modelling approach for the ideal dataset. Comparison of CCS_Ideal_ to the CCS_PARCS_ of the best scoring model from RS (RS CCS), IM (IM CCS), and RG (RG CCS) score function, as well as of the predicted models from AlphaFold2 (AF_with_templates_ CCS) and RoseTTAFold (RF _with_templates_ CCS) for proteins in the ideal dataset.

File Name: Supplementary Data 5

Description: Comparison of CCS predictions with AF and RF for the ideal dataset without templates. Comparison of CCS_Ideal_ to the CCS_PARCS_ of the best scoring model (without the aid of templates) of the predicted models from AlphaFold2 (AF_without_templates_ CCS) and RoseTTAFold (RF_without_templates_ CCS) for proteins in the ideal dataset. The prediction quality of the best scoring models, compared to the native structure (PDB ID), of AF and RF were determined by RMSD and TM-Score (AF_without_templates_ RMSD, AF_without_templates_ TM-Score, RF (RF_without_templates_ RMSD and AF_without_templates_ TM-Score).

File Name: Supplementary Data 6

Description: Description of the experimental dataset. Protein structures from the experimental dataset (PDB ID) with their corresponding sequence length, class, architecture, and domain ID as defined by the protein structure classification database CATH. For this dataset, experimental conditions such as lowest charge state (Charge State) and buffer gas along with modelling protocol (Method) that was utilized to generate decoys per structure are indicated.

Filename: Supplementary Data 7

Description: Benchmarking results of the experimental dataset.. Analysis of best scoring models of protein structures from experimental dataset (PDB ID) to their native structures. These structures were predicted with radius of gyration score function (RG RMSD & RG TM-Score), Rosetta score function (RS RMSD & RS TM-Score), Ion Mobility score function (IM RMSD & IM TM-Score), AlphaFold2 (AF RMSD & AF TM-Score) and RoseTTAFold (RF RMSD & RF TM-Score). Comparison showing the improvement of energy funnel when IM score function was utilized in place of RG (IM_P_near_: RG_P_near_) and RS (IM_P_near_: RS_P_near_) score function.

File Name: Supplementary Data 8

Description: Comparison of CCS predictions with different modelling approach for the experimental dataset. Comparison of CCS_IM_ to the CCS_PARCS_ of the best scoring model from RS (RS CCS), IM (IM CCS), and RG (RG CCS) score function, as well as of the predicted models from AlphaFold2 (AF_with_templates_ CCS) and RoseTTAFold (RF_with_templates_ CCS) for proteins in the experimental dataset.

File Name: Supplementary Data 9

Description:. Subset of proteins (PDB ID) from both the ideal and experimental dataset (Dataset) where ab initio or CM (with non-perfect templates) protocols were used for model generation.

File Name: Supplementary Data 10

Description: Usefulness of IM data for structures with poor quality or no templates in both ideal and experimental dataset. Analysis of best scoring models of protein structures for the subset of proteins where CM (with poor quality templates) and ab initio protocol were run to generate structures. The average RMSD / TM-Score of each of the subsets (ideal, experimental and combined dataset) for the best scoring models selected with the RG score function (RG RMSD & RG TM-Score), RS score function (RS RMSD & RS TM-Score), and the IM score function (IM RMSD & IM TM-Score) are shown.

File Name: Supplementary Data 11

Description: Template information for the ideal dataset. PDB ID (of target and template) and chain ID of templates along with percent sequence coverage and identity as well as RMSD and TM-Score (of template to target) used for CM protocol in Rosetta for proteins in the ideal dataset.

File Name: Supplementary Data 12

Description: Template information for the experimental dataset. PDB ID (of target and template) and chain ID of templates along with percent sequence coverage and identity as well as RMSD and TM-Score (of template to target). The weights column corresponds to how the templates were weighted during model generation with the CM protocol in Rosetta for proteins for the experimental dataset.

File Name: Supplementary Data 13

Description: Recovered structures for selected proteins in the experimental dataset. PDB ID for proteins in experimental dataset. Residues that were added and/or removed from C and N terminal (CT and NT respectively) and the residues are added/removed sequentially from left to right for each terminal.

File Name: Supplementary Data 14

Description: Comparison of AF predictions without templates using two methods described in Supplementary Note 3. Protein structures (PDB ID) were predicted without the aid of templates in AF with two different options that both effectively exclude the use of any templates in the modeling. Option 1 corresponds to predicting structures in AF by setting the max_template_date to 1900-01-01, while option 2 corresponds to modifying the source code to ignore templates. The predicted models are compared by RMSD and TM-Score from option 1 (AF RMSD with option 1 and AF TM-Score with option 1) and option 2 (AF RMSD with option 2 and AF TM-Score with option 2).
