# Supplementary material for: Protein shape sampled by ion mobility mass spectrometry consistently improves protein structure prediction
Source: Nat Commun. 2022 Jul 28;13:4377. doi: 10.1038/s41467-022-32075-9 (PMC9334640; doi:10.1038/s41467-022-32075-9)
Supplement: Supplementary file 17 — Reporting Summary [file 41467_2022_32075_MOESM17_ESM.pdf]

nature portfolio

Corresponding author(s): Steffen Lindert

Last updated by author(s): Jul 13, 2022

Reporting Summary

Nature Portfolio wishes to improve the reproducibility of the work that we publish. This form provides structure for consistency and transparency in reporting. For further information on Nature Portfolio policies, see our Editorial Policies and the Editorial Policy Checklist.

Statistics

For all statistical analyses, confirm that the following items are present in the figure legend, table legend, main text, or Methods section.

n/a	Confirmed	
☐	☒	The exact sample size (*n*) for each experimental group/condition, given as a discrete number and unit of measurement
☒	☐	A statement on whether measurements were taken from distinct samples or whether the same sample was measured repeatedly
☒	☐	The statistical test(s) used AND whether they are one- or two-sided<br>*Only common tests should be described solely by name; describe more complex techniques in the Methods section.*
☒	☐	A description of all covariates tested
☒	☐	A description of any assumptions or corrections, such as tests of normality and adjustment for multiple comparisons
☐	☒	A full description of the statistical parameters including central tendency (e.g. means) or other basic estimates (e.g. regression coefficient) AND variation (e.g. standard deviation) or associated estimates of uncertainty (e.g. confidence intervals)
☒	☐	For null hypothesis testing, the test statistic (e.g. *F*, *t*, *r*) with confidence intervals, effect sizes, degrees of freedom and *P* value noted<br>*Give P values as exact values whenever suitable.*
☒	☐	For Bayesian analysis, information on the choice of priors and Markov chain Monte Carlo settings
☒	☐	For hierarchical and complex designs, identification of the appropriate level for tests and full reporting of outcomes
☒	☐	Estimates of effect sizes (e.g. Cohen's *d*, Pearson's *r*), indicating how they were calculated

Our web collection on statistics for biologists contains articles on many of the points above.

Software and code

Policy information about availability of computer code

Data collection	AlphaFold v2.0.0 (https://github.com/deepmind/alphafold/releases/tag/v2.0.0) and RosettaFold v.1.1.0 (https://github.com/RosettaCommons/RoseTTAFold/releases/tag/v1.1.0) for protein structure prediction. For model quality assessment Voronota v1.22.3149 (https://github.com/kliment-olechnovic/voronota/releases/tag/v1.22.3149), P3CMQA v.1.0.0 (https://github.com/yutake27/P3CMQA) were used. The IM score function for scoring with IM data, PARCS application (v.1.0.0) for Collision Cross Section prediction, are freely available to academic users through the Rosetta Software Suite (v.3.1.3) at https://www.rosettacommons.org/software.
Data analysis	Python v.3.7.3 (https://www.python.org/downloads/release/python-373/), Matplotlib v.3.1.2 (https://matplotlib.org/3.1.1/users/installing.html), PyMOL v.2.0.7 (https://pymol.org/installers/), Blender v.2.8.1 (https://www.blender.org/download/releases/2-81/)

For manuscripts utilizing custom algorithms or software that are central to the research but not yet described in published literature, software must be made available to editors and reviewers. We strongly encourage code deposition in a community repository (e.g. GitHub). See the Nature Portfolio guidelines for submitting code & software for further information.

Data

Policy information about availability of data

All manuscripts must include a data availability statement. This statement should provide the following information, where applicable:

- Accession codes, unique identifiers, or web links for publicly available datasets
- A description of any restrictions on data availability
- For clinical datasets or third party data, please ensure that the statement adheres to our policy

The Protein Data Bank (PDB) was used for all the crystal structure data used in this study. The accession codes of the structures in the ideal dataset are: 1IZ4, 1JMA,

1K7G, 1KB0, 1KTU, 1LI1, 1N7V, 1NLT, 1OKC, 1QHU, 1QQC, 1R3F, 1RNE, 1SUU, 1T9F, 1YNF, 1YU0, 1ZVC, 2F3L, 2FM9, 2GD5, 2ISB, 2JAI, 2P8H, 2QGQ, 2QSD, 2ZZQ, 3A1Y, 3BXO, 3C7X, 3CPW, 3E8T, 3EB7, 3EF6, 3ENI, 3G91, 3GUA, 3KYJ, 3M7M, 3N99, 3ODJ, 3P0Y, 3RST, 3S2O, 3SAE, 3VZ9, 3WPV, 4AI1, 4D6F, 4E7G, 4I1M, 4QMJ, 4XTK, 5KIS, 5LB7, 5MIN, 5U69, 5VSK, 6AZZ, 6S2M. The accession codes of the structures in the experimental dataset are: 1BEB, 1BN1, 1CFD, 1DPX, 1EX3, 1FD3, 1FS3, 1HFX, 1HRC, 1J7N, 1LDS, 1LFG, 1OVA, 1QOY, 1UBQ, 1VXG, 1YTQ, 2MLT, 3INS, 3QYT, 3VWI, 4F5S, 4H2A, 6DAH, 6PTI. Additionally, the 4465 accession codes for the PARCS evaluation dataset can be found in Source Data file. These structures (from all datasets) can be accessed from the PDB [https://www.rcsb.org] with the accession codes provided here and in the Source Data file. The CATH Protein Structure Classification database was used to determine the architectures of proteins and can accessed here: http://www.cathdb.info. The processed simulation data in this work are available through a GitHub repository75 [https://doi.org/10.5281/zenodo.6726418] without any restriction. Additionally, our GitHub repository contains instructions on how this work can be reproduced. Access to raw simulation data (not present in this repository due to size limitation) can be obtained by emailing the corresponding author (lindert.1@osu.edu). The processed simulation data generated in this study are provided in the Supplementary Data files and the Source Data file. Source data are provided with this paper.

Field-specific reporting

Please select the one below that is the best fit for your research. If you are not sure, read the appropriate sections before making your selection.

☒ Life sciences ☐ Behavioural & social sciences ☐ Ecological, evolutionary & environmental sciences

For a reference copy of the document with all sections, see nature.com/documents/nr-reporting-summary-flat.pdf

Life sciences study design

All studies must disclose on these points even when the disclosure is negative.

Sample size	For the experimental dataset, the sample size was determined based on the availability of previously published IM data in the literature. The sample size of ideal dataset was determined based on unique protein architecture types in the CATH database. The sample size of the PARCS evaluation dataset was determined based on the availability of pdbs with sequence identity of less than or equal to 10%, protein sequence length and number of residues (between 40 – 250 residues). The sample size for decoy generation was chosen to be 10,000 based on the rationale that given the size of the proteins, this is a large enough number for Monte Carlo sampling. Additionally we show how the improvement in structure prediction converges as we vary this sampling size from 100 to 10,000. The number of random rotations used in PARCS application is set to 300 as we show how the CCS calculations converges to a stable standard deviation as we vary the random rotations from 100-400.
Data exclusions	No data were excluded.
Replication	Since Rosetta employs a Monte Carlo modeling algorithm, the sampling distribution may not be exactly identically reproduced. But the overall distribution should still more or less be the same. The actual rescoring is reproducible once models have been generated. We have provided a set of 10,000 ab initio structures (zipped) for 3A1Y (PDB-ID from the ideal dataset) as part of example data in the directory "Example_Data" in GitHub (https://doi.org/10.5281/zenodo.6726418). Users can test reproducibility on this dataset. Additionally all commands used to run structure prediction with AlphaFold2, RoseTTAFold, Rosetta (ab initio and comparative modeling) can be found in the directory "Example_Commands" in GitHub (Link: https://doi.org/10.5281/zenodo.6726418).
Randomization	Not applicable; no sample/organism/participant experimental groups were used.
Blinding	Not applicable; no sample/organism/participant experimental groups were used.

Reporting for specific materials, systems and methods

We require information from authors about some types of materials, experimental systems and methods used in many studies. Here, indicate whether each material, system or method listed is relevant to your study. If you are not sure if a list item applies to your research, read the appropriate section before selecting a response.

Materials & experimental systems

n/a	Involved in the study
☒	☐ Antibodies
☒	☐ Eukaryotic cell lines
☒	☐ Palaeontology and archaeology
☒	☐ Animals and other organisms
☒	☐ Human research participants
☒	☐ Clinical data
☒	☐ Dual use research of concern

Methods

n/a	Involved in the study
☒	☐ ChIP-seq
☒	☐ Flow cytometry
☒	☐ MRI-based neuroimaging
